# A Major Genetic Locus in *Trypanosoma brucei* Is a Determinant of Host Pathology

**DOI:** 10.1371/journal.pntd.0000557

**Published:** 2009-12-01

**Authors:** Liam J. Morrison, Andy Tait, Sarah McLellan, Lindsay Sweeney, C. Michael R. Turner, Annette MacLeod

**Affiliations:** 1 Wellcome Trust Centre for Molecular Parasitology, University of Glasgow, Biomedical Research Centre, Glasgow, United Kingdom; 2 Division of Infection and Immunity, Faculty of Biomedical and Life Sciences, University of Glasgow, Biomedical Research Centre, Glasgow, United Kingdom; Yale School of Public Health, United States of America

## Abstract

The progression and variation of pathology during infections can be due to components from both host or pathogen, and/or the interaction between them. The influence of host genetic variation on disease pathology during infections with trypanosomes has been well studied in recent years, but the role of parasite genetic variation has not been extensively studied. We have shown that there is parasite strain-specific variation in the level of splenomegaly and hepatomegaly in infected mice and used a forward genetic approach to identify the parasite loci that determine this variation. This approach allowed us to dissect and identify the parasite loci that determine the complex phenotypes induced by infection. Using the available trypanosome genetic map, a major quantitative trait locus (QTL) was identified on *T. brucei* chromosome 3 (LOD = 7.2) that accounted for approximately two thirds of the variance observed in each of two correlated phenotypes, splenomegaly and hepatomegaly, in the infected mice (named *TbOrg1*). In addition, a second locus was identified that contributed to splenomegaly, hepatomegaly and reticulocytosis (*TbOrg2*). This is the first use of quantitative trait locus mapping in a diploid protozoan and shows that there are trypanosome genes that directly contribute to the progression of pathology during infections and, therefore, that parasite genetic variation can be a critical factor in disease outcome. The identification of parasite loci is a first step towards identifying the genes that are responsible for these important traits and shows the power of genetic analysis as a tool for dissecting complex quantitative phenotypic traits.

## Author Summary

Trypanosomes are single-celled organisms that are transmitted between animal hosts by the tsetse fly. These parasites infect a wide range of mammals and in sub-Saharan Africa are extensively debilitating to livestock, and some species are also able to infect humans causing a disease, sleeping sickness, that is usually fatal unless treated. Some trypanosome strains cause more severe disease than others, and studying these differences may allow the identification of how serious disease is caused. We approached this problem by looking at how differences in disease symptoms (enlarged spleen and liver, and reduced blood cell numbers) that are caused in infections in mice with two strains of *Trypanosoma brucei*, TREU927 and STIB247. These disease manifestations are clinically relevant in human and livestock trypanosome infections. Examining how the symptoms are inherited in infections with offspring of a cross between the two strains allowed the identification of a region of the *T. brucei* genome that contains a gene (or several genes) that contributes significantly towards the enlarged spleen and liver observed in infected mice. This is a first step towards identifying the parasite genes that cause disease in the host (virulence factors), which may provide routes for developing novel therapies against the disease.

## Introduction

Forward genetics provides a powerful tool for analysing phenotypes and identifying genes that are responsible for a number of important traits. The importance of the linkage mapping approach is particularly appropriate for the analysis of phenotypes for which there is no obvious candidate gene, or when more than one gene is predicted to be involved in a phenotype, so called complex traits or quantitative trait loci. Such quantitative traits include disease severity, where there is a range of pathogenesis phenotypes caused by a particular pathogen.

African trypanosomes cause a disease syndrome of high morbidity and mortality across large areas of sub-Saharan Africa in both humans (sleeping sickness) and domesticated animals (Nagana). The parasites are spread by blood-feeding tsetse fly vectors (*Glossina* ssp.), which inject the organisms into the mammalian host's bloodstream where they replicate extracellularly resulting in a chronic wasting condition. Two subspecies of *Trypanosoma brucei*, *T. b. rhodesiense* and *T. b. gambiense*, are able to infect humans causing a disease that is normally fatal and affects approximately 70,000 people per year [1] (although this number is undoubtedly a gross underestimate [2]). In livestock, tens of millions of animals are affected each year with *T. brucei* and the related pathogens *T. congolense* and *T. vivax* [3].

In any host-pathogen relationship, variation in disease outcome can arise from differences between either hosts, pathogens, or both. In trypanosome biology, variation in parasite virulence has been well documented but the genetic basis for this has been largely unexplored. Classically, the two *T. brucei* subspecies have been described as causing different pathology; *T. b. rhodesiense* causes a short, acute disease, while that seen with *T. b. gambiense* is more chronic and less severe [4]. However, the clinical distinctions are unquestionably less clear than textbooks suggest. Laboratory experiments have demonstrated differences in pathogenicity during mouse infections with different strains of *T. b. gambiense* [5],[6], and recently it has been shown that there is markedly different pathology elicited in terms of disease severity and progression between *T. b. rhodesiense* patients in geographically distinct areas in Uganda and Malawi [7],[8]. In the major cattle pathogens, *T. congolense* and *T. vivax*, variation between isolates in clinical pathology are also well documented [9],[10],[11].

Variation in parasite virulence phenotypes that can be directly attributed to trypanosome genetic variation has been experimentally demonstrated in mice in several studies using *T. brucei* and *T. congolense* [12],[13]. Thus, variation is well known and clinically important. The genetic basis for this variation however, has not been investigated. This is in marked contrast to the very considerable effort that has been directed towards dissecting the genetic basis behind variation in pathology (‘trypanotolerance’) attributable to the mammalian host in response to infections with the major veterinary pathogen, *T. congolense* in both mice [14],[15] and cattle [16]. These studies have resulted in the identification of loci in both host systems that contribute to the control of infection.

As all three major African trypanosome species are responsible for a wide range of virulence phenotypes, understanding the genetic determinants of this variation is an essential factor to be integrated into any model of pathogenesis of trypanosomiasis [7],[12],[17]. Therefore, the contribution of the host to the control of disease and the parasite to the pathogenesis must both be examined in order to produce a holistic picture of host-parasite interactions and the survival of the host. Observed differences in virulence between trypanosome strains point to a genetic basis for the spectrum of disease caused in the host and therefore provide a route for identifying the parasite factors that cause disease in the mammalian host. The use of a classical genetic linkage mapping approach, in which the inheritance of phenotypic traits are analysed in progeny of genetic crosses and examined for co-segregation with genetic markers, is a valuable route to identifying genes and loci that has been used for a number traits in pathogenic parasites. This approach is dependent upon the development of a genetic map for each species. Recently, genetic maps have been generated for a number of haploid parasites, for example, *Plasmodium falciparum* [18], *Plasmodium chabaudi* [19], *Toxoplasma gondii* [20], and *Eimeria tenella* [21], opening up the possibility of using classical genetic analysis to identify genes involved in important phenotypes. For example, the identification of protective antigens that enable survival upon exposure to the host immune response in *Eimeria* species [22] and *P. falciparum* [23], a gene that influences the ability to invade red blood cells in *P. falciparum* [24], and genes that confer resistance to chloroquine and quinine in *P. falciparum* [25],[26]. The haploid nature of these organisms means that the contribution of a single allele to a phenotype can be measured in isolation without the complication of an effect of a second allele, allowing for the identification of genes involved in various phenotypes via a linkage mapping approach with remarkably few progeny.

Perhaps the most compelling case for the power of genetic analysis is that of parasite virulence in *T. gondii*, whereby Quantitative Trait Loci (QTL) associated with virulence were identified [20], which directly led to the identification of secreted kinases as the key genes involved in strain specific pathogenesis of toxoplasmosis [27],[28],[29]. Indeed, the relevance of these genes has been confirmed in *T. gondii* field strains [30] and these latter studies elegantly underline the application of the classical genetic approach to identifying genes of large effect in parasites [31],[32]. The development of genetic maps for two sub-species of *T. brucei*, *T. b. brucei* and *T. b. gambiense* [33],[34] provides the potential for using a classical genetic approach in this organism, although the diploid nature of this parasite means that only phenotypes that are heterozygous in the parental strain for which the map was generated can be mapped using F1 progeny.

Pathology in trypanosome-infected hosts is multi-faceted and we have chosen to focus on four indices of pathology; anaemia, reticulocytosis, splenomegaly and hepatomegaly. These traits are all important in both humans and cattle in infections with all three species of trypanosome [11],[35],[36]. Here, we report a genetic analysis of these inherited host pathology traits and show that two, splenomegaly and hepatomegaly, resulted in a highly significant QTL on chromosome 3 of the *T. brucei* genome.

## Methods

### Ethics statement

The maintenance and care of experimental animals complied with the appropriate legislation; the UK Animals (Scientific Procedures) Act, 1986, and with the national and University of Glasgow maintenance and care guidelines.

### Trypanosomes

**Figure 1:**
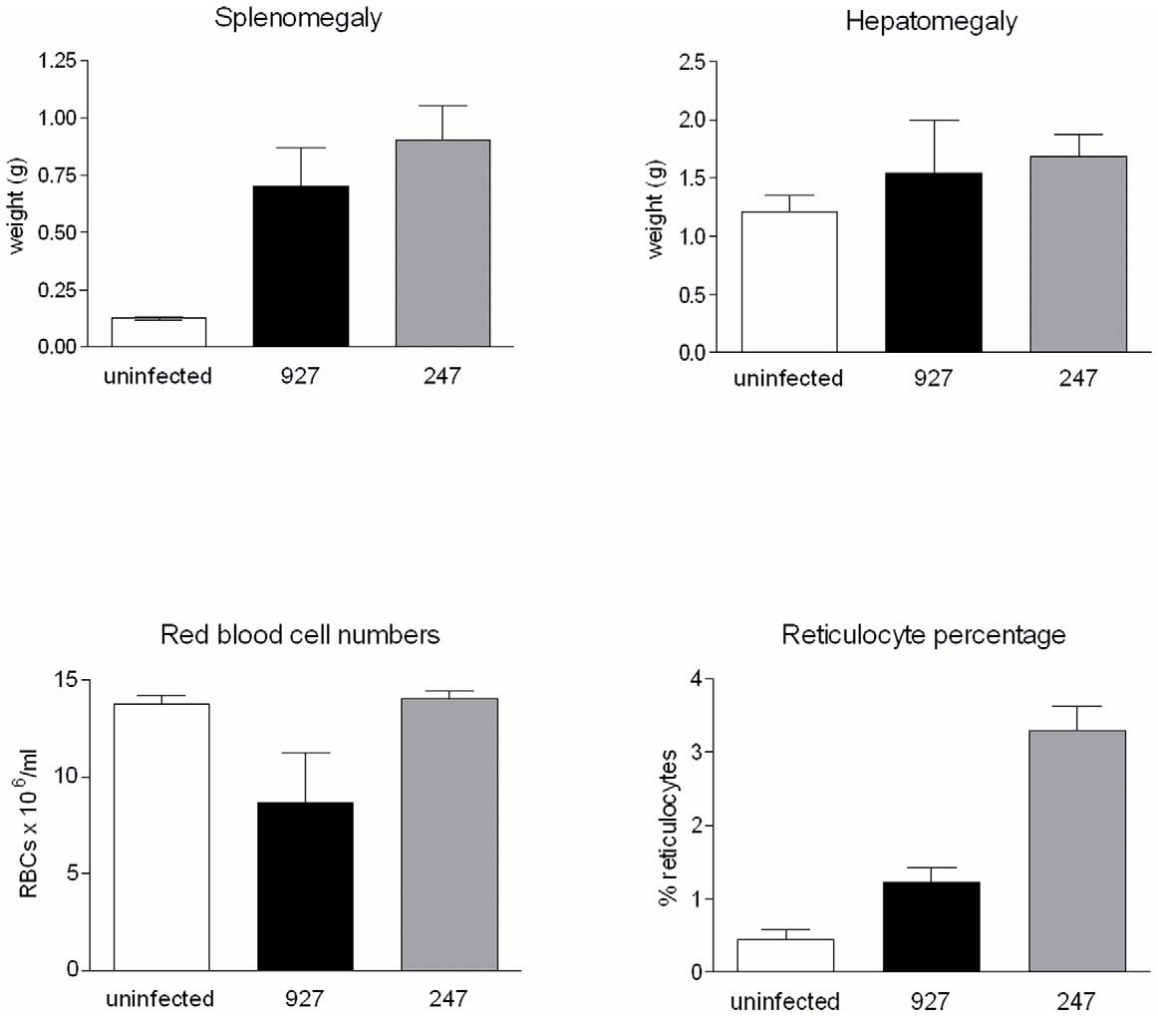
Average phenotype values for infections with STIB247 (grey bar) and TREU927 (black bar) in comparison with uninfected controls (clear bar), for spleen weight, liver weight, red blood cell numbers and percentage reticulocytes. Values are mean±95% confidence interval (n = 5).

A panel of 39 independent F1 *T. brucei* progeny clones had been previously generated from a genetic cross between a strain originally isolated from a tsetse fly (TREU927) and a second isolated from a hartebeeste (STIB247) [37],[38]. Parental and progeny clones were previously genotyped for 182 microsatellite markers [33] and thirty one progeny were used in the current study (Fig. 1). The eight further clones isolated from the TREU927/STIB247 cross are only available at present as the insect life cycle stage procyclic forms and are thus not suitable for *in vivo* mammalian studies. The two parental lines were each transmitted through tsetse flies and recloned (using methods described in [37]) so that their transmission/passage history was very similar to that of the hybrid progeny. All parasites used readily infect rodents and passages in our laboratory are kept to a maximum of six times in mice from tsetse transmission, in order to maintain tsetse transmissibility and minimise selection by growth in rodents [39]. Five mini- and microsatellite markers, all on different chromosomes of *T. brucei*, were used for routine genotyping of all infections (Ch1/MS42, Ch2/PLC, Ch3/IJ15/1, Ch5/JS2 and Ch9/49; for primer sequences and PCR conditions see [33]).

### Animal infections

Parasites were grown from stabilates (cryopreserved in liquid nitrogen) in a donor ICR mouse (Harlan, UK). Parasites in logarithmic growth were harvested from the donor mouse by terminal exsanguination. The trypanosomes were counted in triplicate in an improved Neubauer haemocytometer, and diluted in Carter's Balanced Salt Solution (CBSS) to 1×10^4^ trypanosomes per 0.2 ml inoculum. Parasites were then inoculated via the intraperitoneal route into experimental BALB/c mice (Harlan, UK). Five mice were infected per strain, and an equivalent number of uninfected mice were included as controls. Infections with all of the progeny were completed in nine different batches and, in each case, infections with both parental strains were used to ensure consistency of phenotypes across all of the mouse batches, and to provide a means of minimising batch-to-batch variation (see below). At day 10 post-infection, mice were euthanased. Day 10 was chosen as it was the time point at which the greatest number of pathogenesis phenotypes were significantly different between the parental strains [Morrison et al., submitted].

### Phenotype measurements

We chose to measure two features that can be readily related to known clinical pathology in multiple host species – organomegaly and anaemia. At day 10 post-infection, mice were euthanased and the spleen and liver dissected and weighed. After weighing, the spleens were snap frozen in liquid nitrogen, and stored at −80°C. A FACs based assay was developed to measure anaemia in the mouse model [Morrison et al., submitted]. Briefly, parameters for FACs analysis were determined for each cell population (trypanosomes, red blood cells, reticulocyctes and white blood cells), by assaying each population in isolation with a known number of fluorescent beads, allowing the gating and calculation of cell populations from whole infected blood. For each strain 5 µl blood was collected and added to 2 µl of in CBSS containing 100 U/ml heparin, followed by the addition of 198 µl 1% paraformaldehyde in PBS pH 7.4, mixed thoroughly to ensure no aggregates formed and fixed for 30 minutes at room temperature. To stain reticulocytes, 745 µl thiazole orange (100 ng/ml in PBS pH 7.4) was added to the blood/fixative mixture and allowed to stain for 1 hour in the dark at room temperature [40]. Fluorescent beads (Countbright™ absolute counting beads for flow cytometry, Invitrogen, UK) were prepared by diluting to 1000 beads/µl and ensuring even mixing by sonication. Fifty µl of the bead mixture was then added to the blood/thiazole orange preparation (total volume 1 ml; 50,000 beads/ml). FACS was carried out using a Becton Dickinson FACSCalibur, using detector FL1-H for thiazole orange. Data were analyzed using CellQuest version 3.3. For each sample 10,000 events were counted five times. This assay differs from that recently described by Antoine-Moussiaux et al [41] and is described in full in Morrison et al (submitted).

### Data and QTL analysis

The data for all four phenotypes were normally distributed (D'Agostino-Pearson normality test, p>0.05 in all cases), and this allowed calculation of Pairwise correlations of phenotype data using Pearson correlation coefficient and a two-tailed P-value, and from this a value of r^2^ was generated. For QTL analysis, the mean phenotype value for each progeny clone was calculated as a percentage of the phenotype for the TREU927 parental strain in that particular batch to take batch-to-batch variation into account. QTL analysis was performed using MapManager QTX software [42] for the *T. brucei* genetic map [33]. The variance was calculated from the five biological replicates of each phenotype for each progeny. The Likelihood Ratio Statistic (LRS) significance values were calculated by 1000 random permutations of the phenotypes relative to the genotypes that were intrinsic to the experimental data set (to obtain the equivalent LOD score, the LRS is divided by approximately 4.6). Thresholds of statistical significance (χ^2^ statistic) were calculated from each permutation test for each phenotype. The calculated threshold values were in line with those suggested for genome-wide scan studies [43]. For a simplified explanation of the inheritance model and relevant features of the TREU927 × STIB247 cross see Fig S1.

### Marker generation and update of the genetic map

An additional genetic marker, (TB3/19), was developed for *T. brucei* chromosome 3 based on the allele specific amplification of a 1 kb fragment of DNA of the TREU927 parental strain and the failure to amplify the other TREU927 allele or STIB247 parental alleles, due to a single nucleotide polymorphism in the primer site(s). As a positive control for DNA integrity, primers directed against a second non-polymorphic 1 kb fragment were used (TB3/20), which amplified both TREU927 alleles. This allele-specific PCR (TB3/19) segregated in the progeny and was incorporated into the genetic map. Primer sequences were Ch3/19A TGAGTTCCTCTTGCACTCCC, Ch3/19B TCTTTACGTGTGCGCGCTAGG, Ch3/20A GATGTAATGTCCCTTCGGATTGCG and Ch3/20B GTGTCTTGTAGTTTATGACGGC; PCR conditions were described previously [33]; Genotyping gaps in the original map were also filled (for version 2 of the genetic map, see http://tinyurl.com/trypmap).

## Results

### Phenotype measurements

The trypanosomes strains TREU927 and STIB247 consistently differ in the pathogenesis they induce, including the degree to which they cause anaemia, reticulocytosis, splenomegaly and hepatomegaly (Fig 1). We measured these parameters in nine batches of mice and consistently observed the same pattern with greater splenomegaly and reticulocytosis in 247-infected mice and marked anaemia in 927-infected mice (Fig 1 and Fig S2). Whilst the patterns were consistent, the absolute values showed some batch-to-batch variation and to account for this, subsequent data are presented as percentages of the 927 parental levels. A detailed analysis of hepatomegaly development over multiple time points permitted robust statistical analysis by 2-way ANOVA and showed hepatomegaly to be greater in STIB247-infected mice [Morrison et al, submitted], although the data for day 10 show a smaller difference than was observed for the other three phenotypes (Fig S2).

**Figure 2:**
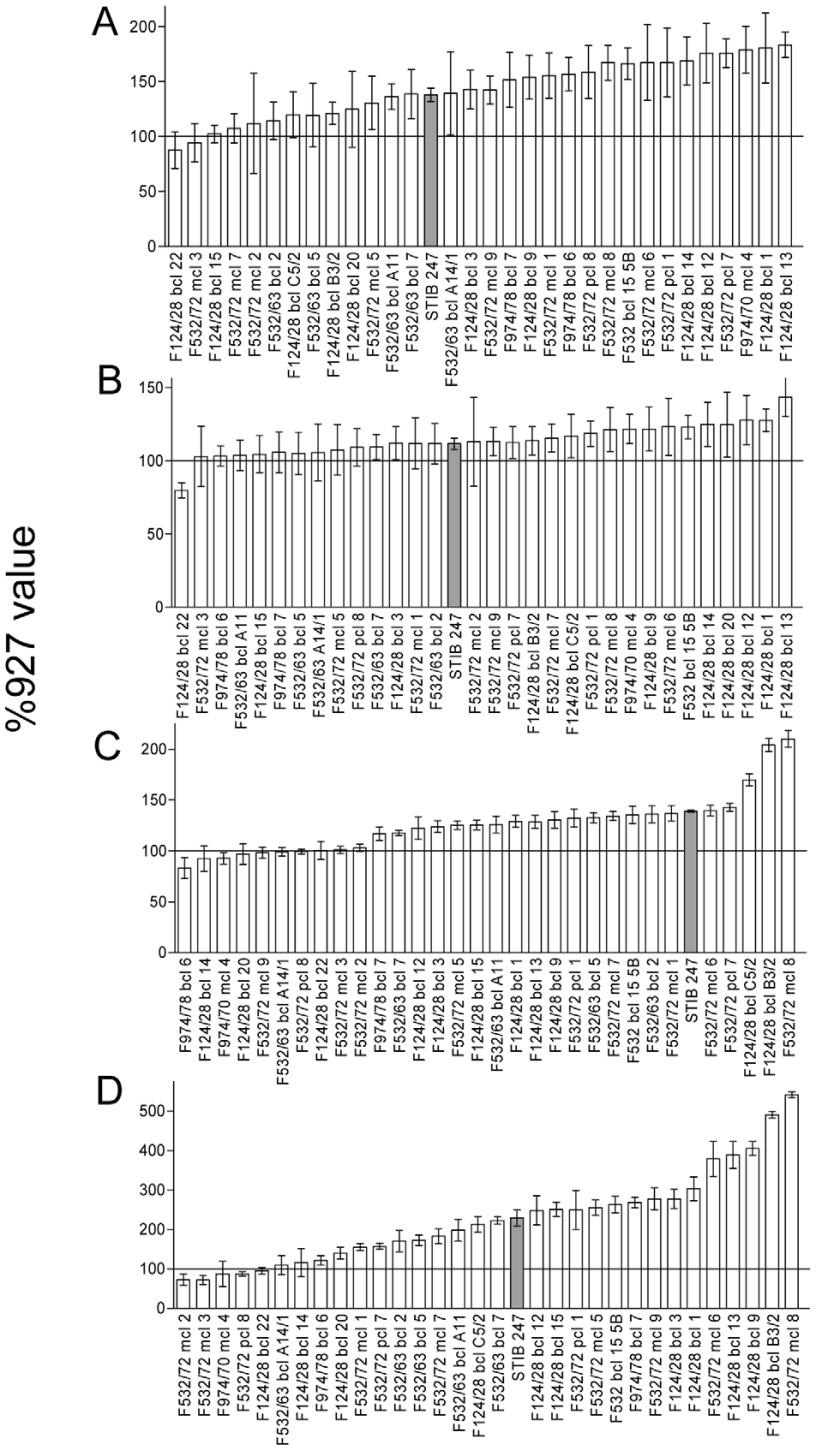
Average phenotype values for infections with the 31 F1 progeny. Measurements taken were A) spleen weight, B) liver weight, C) red blood cell numbers, D) reticulocyte percentage (error bars are 95% confidence interval). Phenotypes are expressed as percentage of the value of the mice infected with TREU927 in the respective batch of infections, and are arranged in ascending order. The mean value for the STIB247 infections is shown (grey bar) and the TREU927 value (100%) is indicated by a solid line. Progeny names are as in the TREU927 × STIB247 genetic map (see [38]).

In order to investigate if there is a genetic basis for this variation in these phenotypes, experimental mouse infections were carried out with 31 independent F1 progeny from a genetic cross between TREU927 and STIB247. The mean data for each phenotype for each progeny clone are illustrated in Figure 2, revealing a continuous distribution for each phenotype. All four phenotypes show marked transgressive segregation with between 10 and 20 hybrids displaying average values outside the parental range. Interestingly, few values fall below that observed for TREU927 and a large number of the progeny display values far greater than either parent. This heterosis was particularly marked for splenomegaly, hepatomegaly and reticulocytosis but less so for red cell numbers. All four phenotypes show classical quantitative inheritance with no clear segregation into different classes in the progeny, but the progeny clones at the top and bottom ends of each distribution show clear and reproducible differences, suggesting that parasite derived genetic determinants contribute to each phenotype.

**Figure 3:**
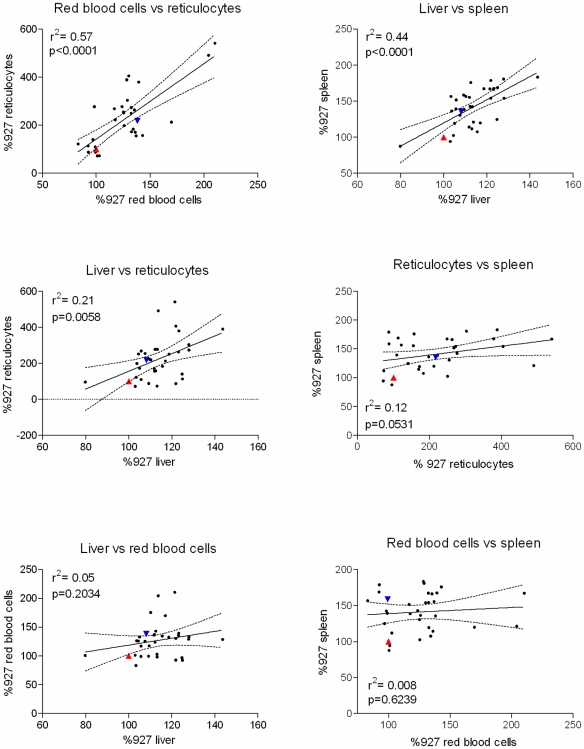
Pairwise correlations between phenotypic data for infections with parents and progeny (significance p<0.05; Pearson correlation coefficient). The p-value and r^2^ value are indicated in each graph, and the line of best fit is also shown (dotted lines indicate the 95% confidence interval). Parental values are shown in each case, with the TREU927 value represented by the red triangle, and the STIB247 value by the inverted blue triangle.

Pairwise correlations were calculated for all phenotypes, revealing three combinations that show significant correlation (Fig 3). The most significant correlation was between red blood cell numbers and percentage reticulocytes, which suggested that 57% of the variance is shared between the two phenotypes. There were also significant correlations between liver and spleen weights (44% of shared variance) and reticulocyte percentage and liver weight (21%).

### Linkage analysis

The relatively continuous distribution of phenotypic data from the F1 progeny in all cases suggests that multiple loci are involved in determining the phenotype. In order to investigate the genetic basis determining these traits a quantitative linkage mapping approach was therefore applied to the data using the available genetic map. The *T. brucei* genetic map created from a TREU927 × STIB247 cross [33] comprising 196 markers spread across the 11 megabase chromosomes (but not covering subtelomeric regions, or mini – and intermediate chromosomes), was used to analyse whether the phenotypes (spleen weight, liver weight, reticulocyte percentage and red blood cell numbers) co-segregated with genetic markers, using QTL analysis.

#### Splenomegaly and hepatomegaly

**Figure 4:**
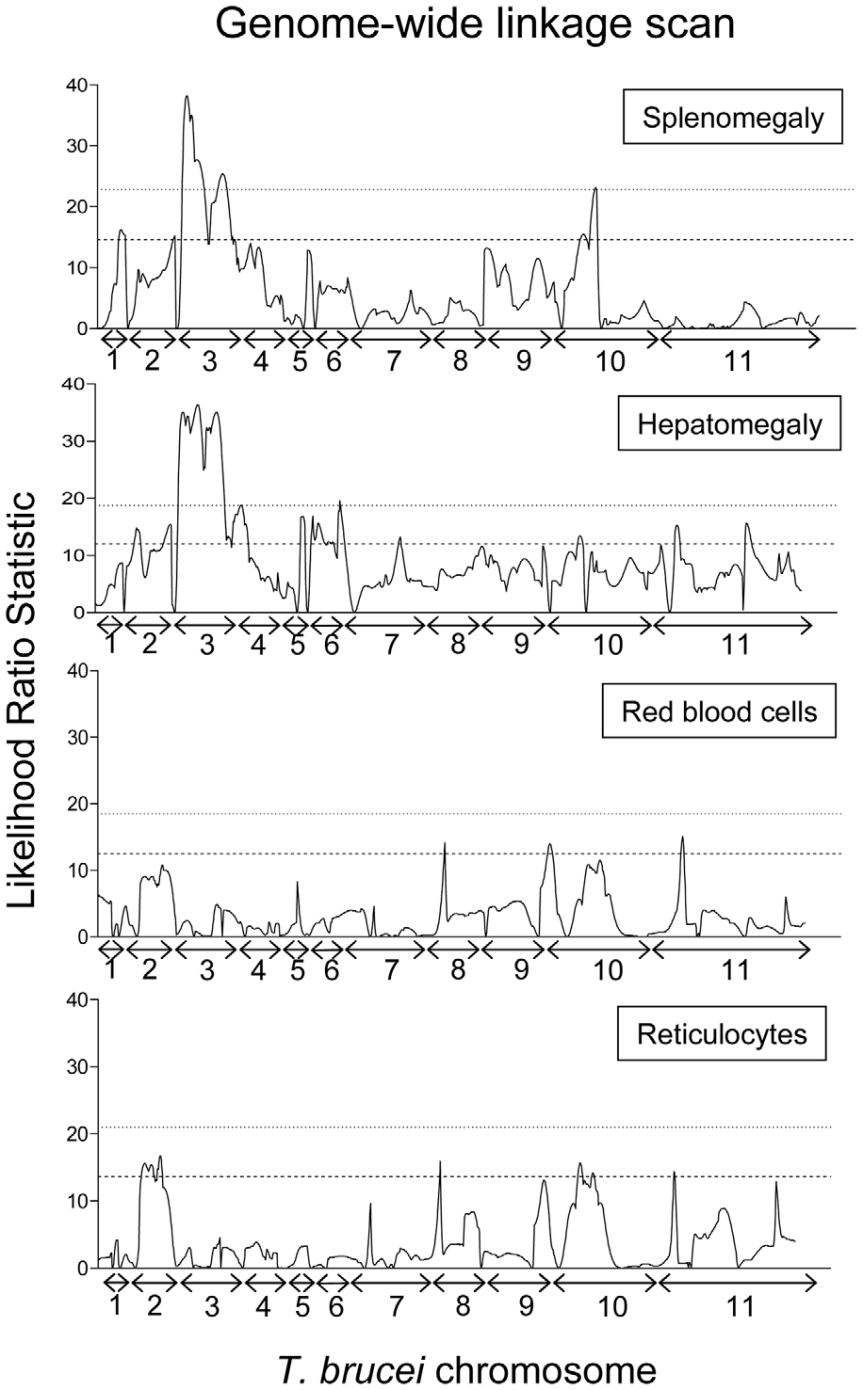
Genome-wide linkage scans for splenomegaly, hepatomegaly, red blood cell numbers and reticulocyte percentage. The graphs show a genome scan of interval mapping for each phenotype from chromosome 1 to 11 of *Trypanosoma brucei*, with the Likelihood Ratio Statistic (probability of linkage of phenotype to genotype; black line) measured in a stepwise manner at 1 cM intervals across the length of the genome; chromosome numbers are shown on the x-axis (the scale on x-axis is in cM). The dashed and dotted lines represent the thresholds of significance (p<0.05) and high significance (P<0.001), respectively, for a genome-wide analysis. For reference, the p<0.05 threshold is approximately equivalent to a LOD score of 3.

**Figure 5:**
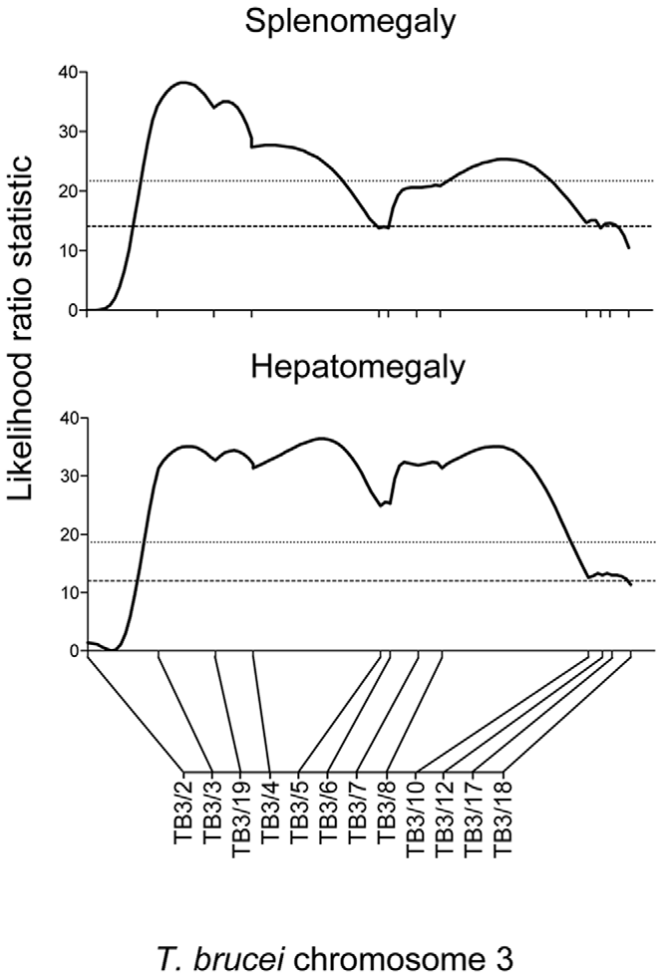
Significant QTLs for splenomegaly and hepatomegaly on chromosome 3 of *Trypanosoma brucei*. The graphs show a chromosome scan of interval mapping for each phenotype, with the Likelihood Ratio Statistic (probability of linkage of phenotype to genotype; black line) measured in a stepwise manner at 1 cM intervals across the length of chromosome 3; chromosome 3 markers are shown on the x-axis (the scale on x-axis is in cM). The dashed and dotted lines represent the thresholds of significance (p<0.05) and high significance (P<0.001), respectively, for a genome-wide analysis.

Two phenotypes, splenomegaly and hepatomegaly, showed evidence for highly significant QTLs, mapping to the same region on chromosome 3 with equivalent LOD scores of 7.2 and 7.5, respectively (Fig 4; Table 1). The hepatomegaly QTL had a significance level of p≤0.001 for a genome-wide analysis, and was estimated to account for approximately 64% of the phenotype variance. The splenomegaly QTL was also highly significant (p≤0.001), and contributed a similar amount (66%) to the phenotype variance. Interval mapping confirmed in both cases that the QTL lies between markers TB3/3 and TB3/10, a region that covers 56.3 cM (physical region between 462529 bp and 1460975 bp, a physical distance of 998446 bp) and which contains 383 genes (Fig 5). For hepatomegaly there is no suggestion of more than one locus involved within the interval of the QTL. However, for splenomegaly although the QTL remains significant between TB3/3 and TB3/10, there are two distinct regions within the QTL showing highly significant linkage (between TB3/3 and TB3/5, and TB3/6 and TB3/10, respectively, Fig 5). This may indicate that there is more than one locus involved in splenomegaly within the context of the QTL (and indeed this could also be the case for hepatomegaly), but this will require further work to fully elucidate. As well as the linkage described above, the hepatomegaly phenotype also shows significant (p<0.05) linkage to minor QTLs on other chromosomes, 2, 4, 5, 6, 7, and 11 and splenomegaly to QTLs on chromosomes 1, 2 and 10 (Fig 3; Table 1).

**Table 1:** Quantitative Trait Loci for splenomegaly, hepatomegaly, red blood cell numbers and reticulocyte percentage, that are significant (p<0.05) and highly significant (p≤0.001) in a genome-wide screen.

Phenotype	Chromosome	Number of marker(s)*	Marker above significance threshold	Significance
Spleen	1	1	TB1/1	p<0.05
	2	5	TB2/16-TB2/20	p<0.05
	**3**	**9**	**TB3/3-TB3/10**	**p≤0.001**
	10	1	TB10/6	p<0.05
Liver	2	4	TB2/22-TB2/6	p<0.05
	2	4	TB2/13-TB2/16	p<0.05
	**3**	**9**	**TB3/3-TB3/10**	**p≤0.001**
	4	3	TB4/16-TB4/11	p<0.05
	5	2	TB5/2-TB5/1	p<0.05
	6	5	TB6/2-TB6/6	p<0.05
	7	2	TB7/1-Tb7/2	p<0.05
	11	2	TB11/3-TB11/4	p<0.05
	11	2	TB11/19-Tb11/20	p<0.05
Red blood cells	10	1	TB10/2	p<0.05
	11	1	TB11/5	p<0.05
Reticulocytes	2	5	TB2/6-TB2/12	p<0.05
	8	1	TB8/5	p<0.05
	11	1	TB11/5	p<0.05

*Number of markers that are significant or highly significant (range of markers is indicated in brackets – for details see http://tinyurl.com/trypmap). Highly significant QTLs are shown in bold.

#### Anaemia (red blood cells and reticulocytosis)

Linkage analysis of percentage reticulocytes revealed significant linkage to three loci on chromosomes 2, 8 and 11, but linkage hinged upon a single marker in the case of chromosomes 8 and 11 and therefore false positive linkage is a possibility. However there are five markers displaying linkage to the same region on chromosome 2, indicative of a genuine QTL (Fig 4; Table 1). Interestingly these overlapping minor QTLs on chromosome 2 appear to be significantly linked to three of the four phenotypes measured in this study: hepatomegaly, splenomegaly and reticulocytosis (Fig 4; Table 1).

For the red blood cell phenotype, two loci were identified as being significantly linked on chromosomes 10 and 11. However, both loci were based on only one marker and so can be considered as either minor loci or a result of a false positive linkage (Fig 4; Table 1).

In summary, we have identified one highly significant locus on *T. brucei* chromosome 3 that accounts for approximately two thirds of the variance observed in splenomegaly and hepatomegaly, which we name *TbOrg1* (for *T. brucei* organomegaly QTL 1). The significance of the QTL on chromosome 3 is reinforced by the fact that both phenotypes show a strong correlation with each other (Fig 3) and link to this region. There is also a smaller, but still significant, locus on chromosome 2 that contributes to strain-specific splenomegaly, hepatomegaly and reticulocytosis, named *TbOrg2*. The importance of *TbOrg2* is enhanced by the fact that three phenotypes are all significantly linked to the locus (Fig 4), contributing approximately 30–35% to each of the phenotypes, and this therefore represents a parasite locus that has a minor but multifactorial effect on pathogenesis in the mouse host. We conducted a secondary screen after fixing *TbOrg1* on chromosome 3, for both splenomegaly and hepatomegaly, but this did not reveal any further significant loci (data not shown).

## Discussion

We have used genetic linkage analysis to investigate a variety of pathogenesis phenotypes in infections with *T. brucei*. Our approach, using genetically distinct parasite strains in one inbred host background effectively means that observed differences in infection profile must originate from parasite genotype differences. Differences caused by host factors also exist, but have been minimised by the use of inbred mice and controlling for any batch-to-batch variation. Our results clearly show that variation in pathology has a parasite determined genetic basis and that using a linkage mapping approach it was possible to identify a region of the genome on chromosome 3 that has a major effect on the gross pathology in mice. This study is an important step, therefore, towards identifying the parasite virulence factors that underlie differential pathogenesis.

The phenotype data in this study do not segregate in a manner that would indicate they are determined by a single gene, unlike that of previous studies in haploid parasites, where traits in progeny could definitively be scored as (for example) virulent, intermediate or non-virulent [20], or drug resistant or sensitive [25]. The phenotypes we have examined are, or undoubtedly can be, the end product of multiple processes, and therefore will have multiple genes contributing to the variance. They are therefore true ‘quantitative traits’, and for this reason QTL analysis was the most appropriate tool to search for linkage between trypanosome genotype and host infection phenotype. The identification of a highly significant QTL, despite the complex nature of the phenotypes, suggests that we have identified a parasite locus that determines a process responsible for a large proportion of the variance observed.

The correlation of liver and spleen weights, and linkage of each to the same region of chromosome 3, means that it is reasonable to assume that the same locus is responsible for the differential pathogenesis in both phenotypes. Whether this is due to a parasite gene product directly influencing both processes individually, or whether the gene product defines the polarisation or sequence in which multiple downstream host and parasite pathways proceed, is one of the questions that remain to be answered. Certainly the spleen and liver are significant organs with respect to the response to infection, and splenomegaly is one of the classical clinical signs observed in experimental cases of animal trypanosomiasis and also in natural cases of animal and human disease [35],[44],[45]. The spleen and liver obviously both have roles with respect to the innate immune response [46] and, with respect to trypanosome infections, the direction in which polarisation of the immune response proceeds seems to determine the progression of disease [47],[48],[49],[50]. We have previously shown that in infections with the two parental strains, pathways of the innate immune response, macrophage polarisation (IL10, RXR/LXR pathways), and alternative macrophage activation are the most significantly differentially regulated [Morrison et al., submitted]. The strain specific pathogenesis is therefore not only due to a difference in magnitude of the same pathways, but distinct pathways. It seems reasonable, therefore, to postulate that there is a parasite gene product that determines this polarisation, and the gene is located within the locus on chromosome 3.

The confidence interval of the major chromosome 3 QTL for splenomegaly and hepatomegaly (*TbOrg1*) includes 383 genes. Of the genes within the QTL, 206 are conserved hypothetical proteins, 109 have function assigned by sequence homology, 62 have a predicted signal peptide, 10 are sequence orphans, 3 are pseudogenes and only 16 have been experimentally characterised (Table S1). Given the number of candidate genes, a fine-mapping approach is required to narrow down the region of linkage and reduce the number of genes to a level where candidates can be experimentally examined. There is scope for this approach, as the QTL interval for both phenotypes contains 12 crossovers in the 31 progeny, and therefore informative markers will further define the linkage boundaries. Fine mapping, in which more informative genetic markers within the QTL region are identified, can be undertaken in a number of ways, the most definitive of which will be to sequence the genomes of the parental strains to identify all single nucleotide polymorphisms within the locus, which can then be used to define the crossover boundaries in the progeny more precisely. There are additional and complementary techniques that will also aid in defining candidate genes, for example; the generation of further progeny in order to incorporate a greater number of informative crossovers in the genetic map and the identification of genes that are heterozygous for 927 (by identifying heterozygous single nucleotide polymorphisms (SNPs) in the relevant chromosome sequence) within the QTL (given the necessity for the gene to be heterozygous to obtain segregation in this cross). Additionally, gene expression data could be used to rule out, for example, those genes only expressed in the procyclic life cycle stage, and inferences of gene function could also be made from the genome sequence data. Similar post-genomic approaches were used successfully to identify *T. gondii* virulence factors, when starting from QTLs spanning regions of a similar magnitude [27]. Additionally, while the approach using F1 progeny clearly allows the identification of dominant heterozygous loci (Fig S1), a limitation is that it will not detect co-dominant or recessive loci. Therefore, using the same approach, but with an F2 cross, would allow the dissection of further loci that are not detectable with the current approach, and provide a more comprehensive picture of the parasite genes contributing to pathogenesis. However, no successful F2 cross has been reported in trypanosomes thus far.

The QTLs identified on chromosome 2 for splenomegaly, hepatomegaly and reticulocytosis (*TbOrg2*) are potentially interesting. In all three cases there are multiple markers linked and the loci of the three phenotypes partially overlap, suggesting that the correlations between the traits (two of which are highly significant and the third approaches significance) may have some causal basis worthy of further investigation. In contrast, there are no QTLs in common for red blood cell numbers and reticulocytosis despite the very strong correlation between these two traits. One explanation for this is that the spleen, and to a lesser extent the liver, are haematopoietic organs, and therefore linked to reticulocyte production. In contrast, the change in red blood cell numbers will largely be a function of the erythrophagocytosis that occurs in early infection [36]. The implication is that, whilst the traits may be linked mechanistically, the parasite determinants that drive organomegaly/reticulocytosis and red blood cell numbers may be different.

In summary, we have demonstrated for the first time that forward genetics provides a powerful tool to map genes in the trypanosome genome that are responsible for causing complex phenotypes in the mouse host. We have defined two loci, one a major locus on Chromosome 3 (*TbOrg1*) that contributes to a significant amount of the variance observed in splenomegaly and hepatomagaly during mouse infections with *T. brucei*, and a second QTL on Chromosome 2 that contributes to splenomegaly, hepatomegaly and reticulocytosis in these infections (*TbOrg2*). Identification of the trypanosome genes that mediate the differential pathogenesis will provide a fundamental insight into the mechanisms by which the parasite causes disease in the mammalian host, and these findings may provide potential for developing therapeutic interventions to alleviate the disease associated with this pathogen.

## Supporting Information

Figure S1 Inheritance model for TREU927 × STIB247 F1 cross. In this simplified model the phenotype is treated as a binary trait determined by a single gene. (Spleen +++ = significantly enlarged spleen, spleen + = slightly enlarged spleen; A = dominant TREU927 allele, a = recessive TREU927 allele, x = STIB247 allele). The cross will allow the detection of traits encoded by a dominant heterozygous allele in TREU927. The genome of STIB247 is predominantly homozygous, with 94% of markers shown to be homozygous [33]. Therefore, the map is constructed based on segregation of alleles in the F1 progeny for loci heterozygous in the TREU 927 parent only. In order to detect co-dominant or recessive alleles, it will be necessary to generate a panel of F2 progeny. For segregation data see http://tinyurl.com/trypmap.
(0.15 MB TIF)

Figure S2 Phenotype values for mice infected with 927 and 247, and uninfected mice for the nine batches of mice; A = splenomegaly, B = hepatomegaly, C = red blood cell counts and D = percentage reticulocytes. In each batch (1–9), clear bar indicates uninfected mice, black bar indicates 927-infected mice and the grey bar indicates 247-infected mice. Values are mean ±95% CI (n = 5).
(0.66 MB TIF)

Table S1 Gene ID, location and annotation for the 383 genes within the QTLs for splenomegaly and hepatomegaly.
(0.41 MB DOC)

## References

[1] WHO (2006). Human African trypanosomiasis (sleeping sickness): epidemiological update.. Wkly Epidemiol Rec.

[2] Fevre EM, Wissmann BV, Welburn SC, Lutumba P (2008). The burden of Human African Trypanosomiasis.. PLoS Negl Trop Dis.

[3] Delespaux V, Geysen D, Van den Bossche P, Geerts S (2008). Molecular tools for the rapid detection of drug resistance in animal trypanosomes.. Trends Parasitol.

[4] Barrett MP, Burchmore RJ, Stich A, Lazzari JO, Frasch AC (2003). The trypanosomiases.. Lancet.

[5] Beckers A, Wery M, Van Marck E, Gigase P (1981). Experimental infections of laboratory rodents with recently isolated stocks of *Trypanosoma brucei gambiense*. 1. Parasitological investigations.. Z Parasitenkd.

[6] Holzmuller P, Biron DG, Courtois P, Koffi M, Bras-Goncalves R (2008). Virulence and pathogenicity patterns of *Trypanosoma brucei gambiense* field isolates in experimentally infected mouse: differences in host immune response modulation by secretome and proteomics.. Microbes Infect.

[7] MacLean L, Chisi JE, Odiit M, Gibson WC, Ferris V (2004). Severity of human african trypanosomiasis in East Africa is associated with geographic location, parasite genotype, and host inflammatory cytokine response profile.. Infect Immun.

[8] MacLean L, Odiit M, MacLeod A, Morrison L, Sweeney L (2007). Spatially and Genetically Distinct African Trypanosome Virulence Variants Defined by Host Interferon- gamma Response.. J Infect Dis.

[9] Bengaly Z, Sidibe I, Boly H, Sawadogo L, Desquesnes M (2002). Comparative pathogenicity of three genetically distinct *Trypanosoma congolense*-types in inbred Balb/c mice.. Vet Parasitol.

[10] Bengaly Z, Sidibe I, Ganaba R, Desquesnes M, Boly H (2002). Comparative pathogenicity of three genetically distinct types of *Trypanosoma congolense* in cattle: clinical observations and haematological changes.. Vet Parasitol.

[11] Gardiner PR (1989). Recent studies of the biology of *Trypanosoma vivax*.. Adv Parasitol.

[12] Masumu J, Marcotty T, Geysen D, Geerts S, Vercruysse J (2006). Comparison of the virulence of *Trypanosoma congolense* strains isolated from cattle in a trypanosomiasis endemic area of eastern Zambia.. Int J Parasitol.

[13] Turner CM, Aslam N, Dye C (1995). Replication, differentiation, growth and the virulence of *Trypanosoma brucei* infections.. Parasitology.

[14] Kemp SJ, Iraqi F, Darvasi A, Soller M, Teale AJ (1997). Localization of genes controlling resistance to trypanosomiasis in mice.. Nat Genet.

[15] Iraqi F, Clapcott SJ, Kumari P, Haley CS, Kemp SJ (2000). Fine mapping of trypanosomiasis resistance loci in murine advanced intercross lines.. Mamm Genome.

[16] Hanotte O, Ronin Y, Agaba M, Nilsson P, Gelhaus A (2003). Mapping of quantitative trait loci controlling trypanotolerance in a cross of tolerant West African N'Dama and susceptible East African Boran cattle.. Proc Natl Acad Sci U S A.

[17] Gardiner PR, Assoku RK, Whitelaw DD, Murray M (1989). Haemorrhagic lesions resulting from *Trypanosoma vivax* infection in Ayrshire cattle.. Vet Parasitol.

[18] Su X, Ferdig MT, Huang Y, Huynh CQ, Liu A (1999). A genetic map and recombination parameters of the human malaria parasite *Plasmodium falciparum*.. Science.

[19] Martinelli A, Hunt P, Fawcett R, Cravo PV, Walliker D (2005). An AFLP-based genetic linkage map of *Plasmodium chabaudi chabaudi*.. Malar J.

[20] Su C, Howe DK, Dubey JP, Ajioka JW, Sibley LD (2002). Identification of quantitative trait loci controlling acute virulence in *Toxoplasma gondii*.. Proc Natl Acad Sci U S A.

[21] Shirley MW, Harvey DA (2000). A genetic linkage map of the apicomplexan protozoan parasite *Eimeria tenella*.. Genome Res.

[22] Blake DP, Shirley MW, Smith AL (2006). Genetic identification of antigens protective against coccidia.. Parasite Immunol.

[23] Martinelli A, Cheesman S, Hunt P, Culleton R, Raza A (2005). A genetic approach to the de novo identification of targets of strain-specific immunity in malaria parasites.. Proc Natl Acad Sci U S A.

[24] Hayton K, Gaur D, Liu A, Takahashi J, Henschen B (2008). Erythrocyte binding protein PfRH5 polymorphisms determine species-specific pathways of *Plasmodium falciparum* invasion.. Cell Host Microbe.

[25] Ferdig MT, Cooper RA, Mu J, Deng B, Joy DA (2004). Dissecting the loci of low-level quinine resistance in malaria parasites.. Mol Microbiol.

[26] Rohrbach P, Sanchez CP, Hayton K, Friedrich O, Patel J (2006). Genetic linkage of *pfmdr1* with food vacuolar solute import in *Plasmodium falciparum*.. Embo J.

[27] Saeij JP, Boyle JP, Coller S, Taylor S, Sibley LD (2006). Polymorphic secreted kinases are key virulence factors in toxoplasmosis.. Science.

[28] Taylor S, Barragan A, Su C, Fux B, Fentress SJ (2006). A secreted serine-threonine kinase determines virulence in the eukaryotic pathogen *Toxoplasma gondii*.. Science.

[29] Saeij JP, Coller S, Boyle JP, Jerome ME, White MW (2007). *Toxoplasma* co-opts host gene expression by injection of a polymorphic kinase homologue.. Nature.

[30] Khan A, Taylor S, Ajioka JW, Rosenthal BM, Sibley LD (2009). Selection at a single locus leads to widespread expansion of *Toxoplasma gondii* lineages that are virulent in mice.. PLoS Genet.

[31] Sibley LD (2009). Development of forward genetics in *Toxoplasma gondii*.. Int J Parasitol.

[32] Sibley LD, Qiu W, Fentress S, Taylor SJ, Khan A (2009). Forward genetics in *Toxoplasma gondii* reveals a family of rhoptry kinases that mediates pathogenesis.. Eukaryot Cell.

[33] MacLeod A, Tweedie A, McLellan S, Taylor S, Hall N (2005). The genetic map and comparative analysis with the physical map of *Trypanosoma brucei*.. Nucleic Acids Res.

[34] Cooper A, Tait A, Sweeney L, Tweedie A, Morrison L (2008). Genetic analysis of the human infective trypanosome *Trypanosoma brucei gambiense*: chromosomal segregation, crossing over, and the construction of a genetic map.. Genome Biol.

[35] Pentreath VW, Kennedy PGE, Maudlin I, Holmes PH, Miles MA (2004). Pathogenesis of Human African Trypanosomiasis.. The Trypanosomiases.

[36] Noyes HA, Alimohammadian MH, Agaba M, Brass A, Fuchs H (2009). Mechanisms controlling anaemia in *Trypanosoma congolense* infected mice.. PLoS ONE.

[37] Turner CM, Sternberg J, Buchanan N, Smith E, Hide G (1990). Evidence that the mechanism of gene exchange in *Trypanosoma brucei* involves meiosis and syngamy.. Parasitology.

[38] MacLeod A, Tweedie A, McLellan S, Hope M, Taylor S (2005). Allelic segregation and independent assortment in *T. brucei* crosses: proof that the genetic system is Mendelian and involves meiosis.. Mol Biochem Parasitol.

[39] Turner CM (1990). The use of experimental artefacts in African trypanosome research.. Parasitol Today.

[40] Weiss DJ (2002). Application of flow cytometric techniques to veterinary clinical hematology.. Vet Clin Pathol.

[41] Antoine-Moussiaux N, Saerens D, Desmecht D (2008). Flow cytometric enumeration of parasitaemia and haematologic changes in *Trypanosoma*-infected mice.. Acta Trop.

[42] Manly KF, Cudmore RH, Meer JM (2001). Map Manager QTX, cross-platform software for genetic mapping.. Mamm Genome.

[43] Lander E, Kruglyak L (1995). Genetic dissection of complex traits: guidelines for interpreting and reporting linkage results.. Nat Genet.

[44] Naessens J (2006). Bovine trypanotolerance: A natural ability to prevent severe anaemia and haemophagocytic syndrome?. Int J Parasitol.

[45] Taylor K, Authie E, Maudlin I, Holmes PH, Miles MA (2004). Pathogenesis of Animal Trypanosomiasis.. The Trypanosomiases.

[46] Engwerda CR, Beattie L, Amante FH (2005). The importance of the spleen in malaria.. Trends Parasitol.

[47] Guilliams M, Oldenhove G, Noel W, Herin M, Brys L (2007). African trypanosomiasis: naturally occurring regulatory T cells favor trypanotolerance by limiting pathology associated with sustained type 1 inflammation.. J Immunol.

[48] Bosschaerts T, Guilliams M, Noel W, Herin M, Burk RF (2008). Alternatively activated myeloid cells limit pathogenicity associated with African trypanosomiasis through the IL-10 inducible gene selenoprotein P.. J Immunol.

[49] Guilliams M, Movahedi K, Bosschaerts T, VandenDriessche T, Chuah MK (2009). IL-10 dampens TNF/inducible nitric oxide synthase-producing dendritic cell-mediated pathogenicity during parasitic infection.. J Immunol.

[50] Namangala B, de Baetselier P, Brijs L, Stijlemans B, Noel W (2000). Attenuation of *Trypanosoma brucei* is associated with reduced immunosuppression and concomitant production of Th2 lymphokines.. J Infect Dis.

