# Supplementary figures and images for: A Major Genetic Locus in Trypanosoma brucei Is a Determinant of Host Pathology
Source: PLoS Negl Trop Dis. 2009 Dec 1;3(12):e557. doi: 10.1371/journal.pntd.0000557 (PMC2780326; doi:10.1371/journal.pntd.0000557)

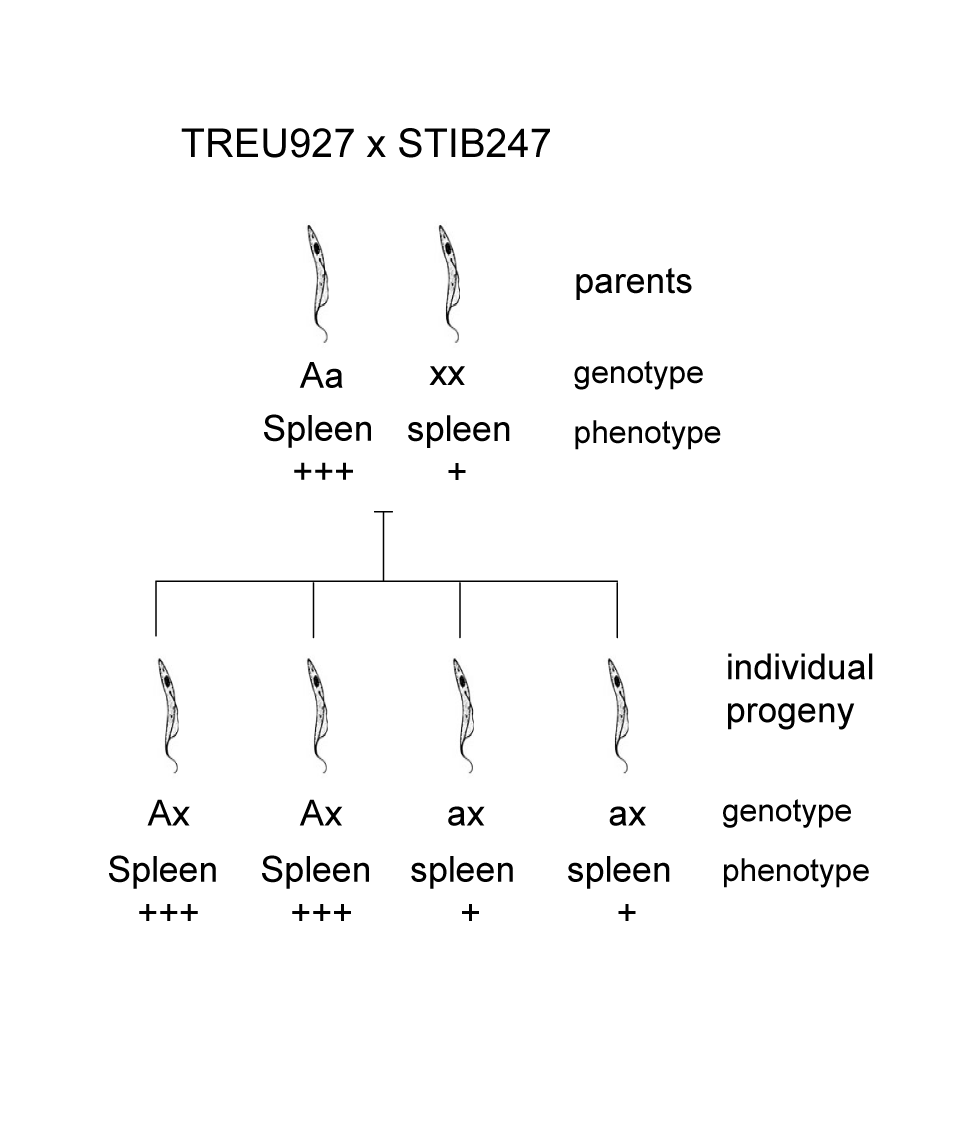

Supplement: Figure S1 — Inheritance model for TREU927 × STIB247 F1 cross. In this simplified model the phenotype is treated as a binary trait determined by a single gene. (Spleen +++ = significantly enlarged spleen, spleen + = slightly enlarged spleen; A = dominant TREU927 allele, a = recessive TREU927 allele, x = STIB247 allele). The cross will allow the detection of traits encoded by a dominant heterozygous allele in TREU927. The genome of STIB247 is predominantly homozygous, with 94% of markers shown to be homozygous [33]. Therefore, the map is constructed based on segregation of alleles in the F1 progeny for loci heterozygous in the TREU 927 parent only. In order to detect co-dominant or recessive alleles, it will be necessary to generate a panel of F2 progeny. For segregation data see http://tinyurl.com/trypmap. (0.15 MB TIF) [file pntd.0000557.s001.tif]

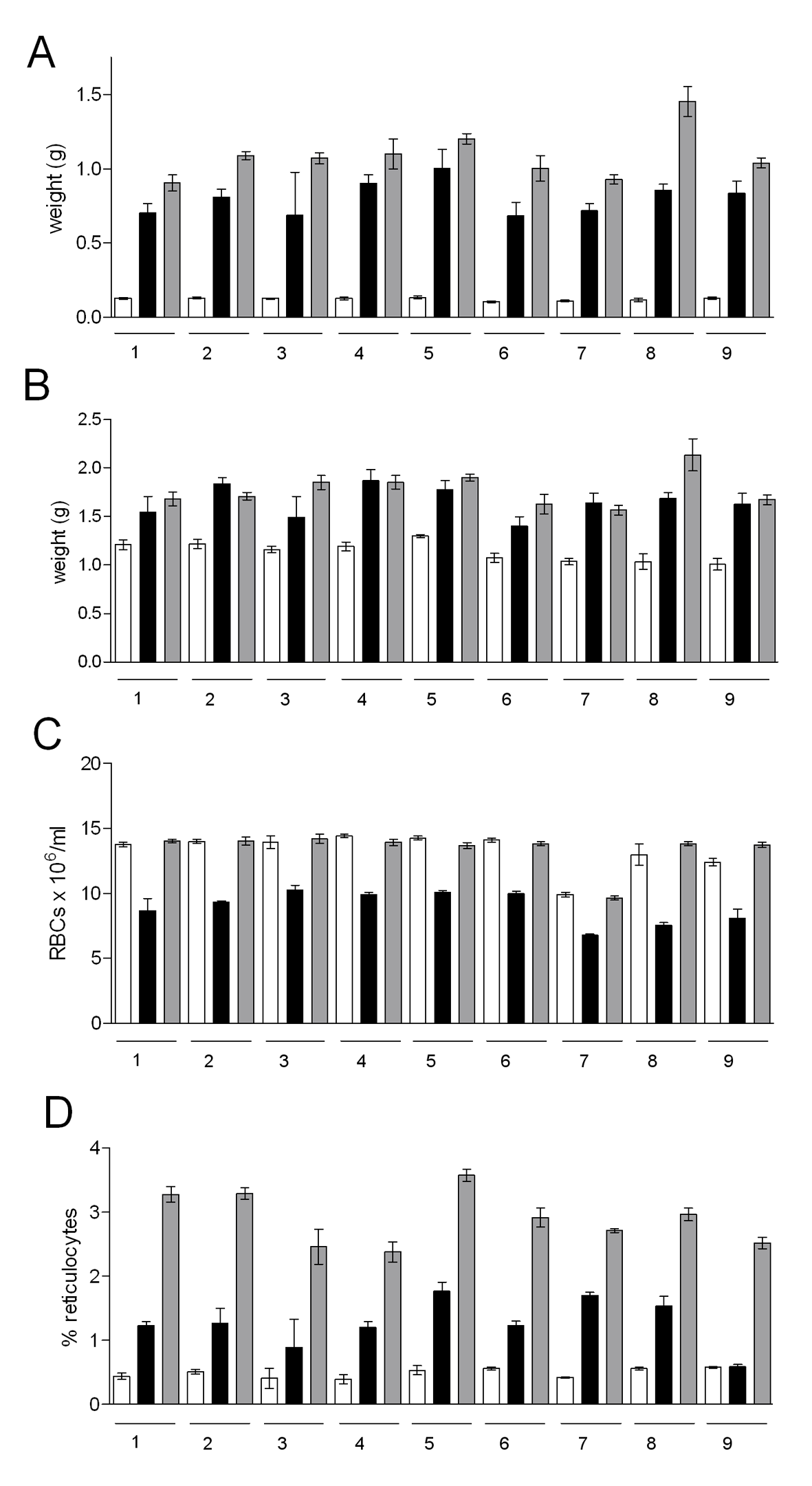

Supplement: Figure S2 — Phenotype values for mice infected with 927 and 247, and uninfected mice for the nine batches of mice; A = splenomegaly, B = hepatomegaly, C = red blood cell counts and D = percentage reticulocytes. In each batch (1–9), clear bar indicates uninfected mice, black bar indicates 927-infected mice and the grey bar indicates 247-infected mice. Values are mean ±95% CI (n = 5). (0.66 MB TIF) [file pntd.0000557.s002.tif]
