# Supplementary material for: A Major Genetic Locus in Trypanosoma brucei Is a Determinant of Host Pathology
Source: PLoS Negl Trop Dis. 2009 Dec 1;3(12):e557. doi: 10.1371/journal.pntd.0000557 (PMC2780326; doi:10.1371/journal.pntd.0000557)
Supplement: Table S1 — Gene ID, location and annotation for the 383 genes within the QTLs for splenomegaly and hepatomegaly. (0.41 MB DOC) [file pntd.0000557.s003.doc]

Table S1. Gene ID, location and annotation for the 383 genes within the QTLs for splenomegaly and hepatomegaly.

| **Gene ID** | **Gene location** | **Product description** |
| --- | --- | --- |
| Tb927.3.3560 | Tb927_03_v4: 1,001,768 - 1,003,642 | hypothetical protein, conserved |
| Tb927.3.3570 | Tb927_03_v4: 1,005,410 - 1,005,898 | hypothetical protein, conserved |
| Tb927.3.3580 | Tb927_03_v4: 1,006,678 - 1,008,999 | lipophosphoglycan biosynthetic protein, putative |
| Tb927.3.3590 | Tb927_03_v4: 1,010,115 - 1,012,133 | U3 small nucleolar ribonucleoprotein protein MPP10, putative |
| Tb927.3.3600 | Tb927_03_v4: 1,014,002 - 1,014,478 | hypothetical protein |
| Tb927.3.3610 | Tb927_03_v4: 1,014,846 - 1,015,931 | peroxisomal targeting signal type 2 receptor, putative |
| Tb927.3.3620 | Tb927_03_v4: 1,016,724 - 1,017,194 | hypothetical protein, conserved |
| Tb927.3.3630 | Tb927_03_v4: 1,018,362 - 1,019,186 | elongation factor Ts, putative |
| Tb927.3.3640 | Tb927_03_v4: 1,020,366 - 1,022,621 | FG-GAP repeat protein, putative; intergrin alpha chain protein, putative |
| Tb927.3.3650 | Tb927_03_v4: 1,022,761 - 1,024,569 | hypothetical protein, conserved |
| Tb927.3.3660 | Tb927_03_v4: 1,025,518 - 1,026,312 | hypothetical protein, conserved |
| Tb927.3.3670 | Tb927_03_v4: 1,027,144 - 1,029,015 | RNA-binding protein, putative |
| Tb927.3.3680 | Tb927_03_v4: 1,029,507 - 1,029,920 | hypothetical protein, conserved |
| Tb927.3.3690 | Tb927_03_v4: 1,030,636 - 1,032,282 | flagellar radial spoke protein-like, putative |
| Tb927.3.3700 | Tb927_03_v4: 1,033,431 - 1,033,898 | hypothetical protein |
| Tb927.3.3710 | Tb927_03_v4: 1,034,040 - 1,034,816 | hypothetical protein, conserved |
| Tb927.3.3720 | Tb927_03_v4: 1,035,275 - 1,035,634 | Golgi vesicular membrane trafficking protein, putative |
| Tb927.3.3730 | Tb927_03_v4: 1,037,620 - 1,043,157 | ABC transporter, putative |
| Tb927.3.3740 | Tb927_03_v4: 1,044,183 - 1,046,048 | hypothetical protein, conserved |
| Tb927.3.3750 | Tb927_03_v4: 1,047,551 - 1,048,081 | hypothetical protein, conserved |
| Tb927.3.3760 | Tb927_03_v4: 1,050,464 - 1,050,898 | tryparedoxin |
| Tb927.3.3770 | Tb927_03_v4: 1,051,543 - 1,052,403 | hypothetical protein, conserved |
| Tb927.3.3780 | Tb927_03_v4: 1,053,583 - 1,054,017 | tryparedoxin |
| Tb927.3.3790 | Tb927_03_v4: 1,054,662 - 1,055,522 | hypothetical protein, conserved |
| Tb927.3.3800 | Tb927_03_v4: 1,056,000 - 1,059,074 | hypothetical protein, conserved |
| Tb927.3.3810 | Tb927_03_v4: 1,059,495 - 1,060,571 | hypothetical protein, conserved |
| Tb927.3.3820 | Tb927_03_v4: 1,061,367 - 1,062,587 | hypothetical protein, conserved |
| Tb927.3.3830 | Tb927_03_v4: 1,063,644 - 1,064,780 | hypothetical protein, conserved |
| Tb927.3.3840 | Tb927_03_v4: 1,065,182 - 1,066,234 | hypothetical protein, conserved |
| Tb927.3.3850 | Tb927_03_v4: 1,066,556 - 1,070,719 | hypothetical protein, conserved |
| Tb927.3.3860 | Tb927_03_v4: 1,071,344 - 1,075,192 | lipase, putative; triacylglycerol lipase, putative |
| Tb927.3.3870 | Tb927_03_v4: 1,076,071 - 1,079,769 | lipase domain protein, putative |
| Tb927.3.3880 | Tb927_03_v4: 1,080,873 - 1,081,286 | hypothetical protein, conserved |
| Tb927.3.3890 | Tb927_03_v4: 1,081,994 - 1,082,956 | hypothetical protein, conserved |
| Tb927.3.3900 | Tb927_03_v4: 1,084,019 - 1,085,884 | carnitine O-palmitoyltransferase II, putative |
| Tb927.3.3910 | Tb927_03_v4: 1,088,132 - 1,089,199 | hypothetical protein, conserved |
| Tb927.3.3920 | Tb927_03_v4: 1,089,619 - 1,090,761 | serine/threonine-protein kinase, putative |
| Tb927.3.3930 | Tb927_03_v4: 1,092,097 - 1,093,377 | RNA-binding protein, putative |
| Tb927.3.3940 | Tb927_03_v4: 1,097,682 - 1,099,463 | RNA-binding protein, putative |
| Tb927.3.3950 | Tb927_03_v4: 1,107,571 - 1,109,223 | hypothetical protein, conserved |
| Tb927.3.3960 | Tb927_03_v4: 1,110,465 - 1,111,598 | RNA-binding protein, putative |
| Tb927.3.3970 | Tb927_03_v4: 1,114,192 - 1,115,085 | hypothetical protein, conserved |
| Tb927.3.3980 | Tb927_03_v4: 1,116,802 - 1,118,163 | hypothetical protein, conserved |
| Tb927.3.3990 | Tb927_03_v4: 1,119,194 - 1,120,510 | RNA-editing complex protein; KREPB6 |
| Tb927.3.4000 | Tb927_03_v4: 1,121,110 - 1,121,589 | clathrin coat assembly protein AP19, putative |
| Tb927.3.4010 | Tb927_03_v4: 1,124,707 - 1,125,297 | hypothetical protein |
| Tb927.3.4020 | Tb927_03_v4: 1,127,210 - 1,134,148 | phosphatidylinositol 4-kinase alpha, putative |
| Tb927.3.4030 | Tb927_03_v4: 1,135,385 - 1,139,041 | hypothetical protein, conserved |
| Tb927.3.4040 | Tb927_03_v4: 1,140,259 - 1,142,118 | hypothetical protein, conserved |
| Tb927.3.4050 | Tb927_03_v4: 1,142,656 - 1,143,153 | hypothetical protein, conserved |
| Tb927.3.4060 | Tb927_03_v4: 1,143,431 - 1,146,001 | hypothetical protein, conserved |
| Tb927.3.4070 | Tb927_03_v4: 1,148,265 - 1,150,022 | hypothetical protein, conserved |
| Tb927.3.4080 | Tb927_03_v4: 1,150,562 - 1,152,277 | hypothetical protein, conserved |
| Tb927.3.4090 | Tb927_03_v4: 1,152,862 - 1,154,613 | hypothetical protein, conserved |
| Tb927.3.4100 | Tb927_03_v4: 1,155,211 - 1,156,938 | hypothetical protein, conserved |
| Tb927.3.4110 | Tb927_03_v4: 1,157,502 - 1,159,289 | hypothetical protein, conserved |
| Tb927.3.4120 | Tb927_03_v4: 1,160,791 - 1,162,863 | hypothetical protein, conserved |
| Tb927.3.4130 | Tb927_03_v4: 1,163,487 - 1,164,068 | hypothetical protein, conserved |
| Tb927.3.4140 | Tb927_03_v4: 1,164,410 - 1,166,095 | hypothetical protein, conserved |
| Tb927.3.4150 | Tb927_03_v4: 1,167,017 - 1,168,369 | hypothetical protein, conserved |
| Tb927.3.4160 | Tb927_03_v4: 1,170,160 - 1,171,125 | hypothetical protein, conserved |
| Tb927.3.4170 | Tb927_03_v4: 1,171,660 - 1,172,019 | hypothetical protein, conserved |
| Tb927.3.4180 | Tb927_03_v4: 1,173,210 - 1,174,436 | hypothetical protein |
| Tb927.3.4190 | Tb927_03_v4: 1,176,215 - 1,178,404 | endosomal integral membrane protein, putative |
| Tb927.3.4200 | Tb927_03_v4: 1,178,789 - 1,179,454 | hypothetical protein, conserved |
| Tb927.3.4210 | Tb927_03_v4: 1,179,908 - 1,185,337 | hypothetical protein, conserved |
| Tb927.3.4220 | Tb927_03_v4: 1,186,243 - 1,188,843 | Zn-finger domain protein, putative |
| Tb927.3.4230 | Tb927_03_v4: 1,190,317 - 1,194,483 | subtilisin-like serine peptidase |
| Tb927.3.4240 | Tb927_03_v4: 1,195,175 - 1,195,777 | thioredoxin, putative |
| Tb927.3.4250 | Tb927_03_v4: 1,196,904 - 1,197,572 | hypothetical protein, conserved |
| Tb927.3.4260 | Tb927_03_v4: 1,198,788 - 1,199,894 | hypothetical protein, conserved |
| Tb927.3.4270 | Tb927_03_v4: 1,202,789 - 1,205,539 | hypothetical protein, conserved |
| Tb927.3.4280 | Tb927_03_v4: 1,206,170 - 1,208,368 | mismatch repair protein MSH5, putative |
| Tb927.3.4290 | Tb927_03_v4: 1,208,864 - 1,210,633 | 73 kDa paraflagellar rod protein; PFR1 |
| Tb927.3.4300 | Tb927_03_v4: 1,211,027 - 1,212,796 | 73 kDa paraflagellar rod protein; PFR1 |
| Tb927.3.4310 | Tb927_03_v4: 1,213,190 - 1,214,959 | 73 kDa paraflagellar rod protein; PFR1 |
| Tb927.3.4320 | Tb927_03_v4: 1,215,353 - 1,217,122 | 73 kDa paraflagellar rod protein; PFR1 |
| Tb927.3.4330 | Tb927_03_v4: 1,217,516 - 1,219,285 | 73 kDa paraflagellar rod protein; PFR1 |
| Tb927.3.4340 | Tb927_03_v4: 1,219,486 - 1,220,559 | diphthamide synthesis protein, putative |
| Tb927.3.4350 | Tb927_03_v4: 1,220,869 - 1,223,262 | hypothetical protein, conserved |
| Tb927.3.4360 | Tb927_03_v4: 1,223,453 - 1,223,725 | 40S ribosomal protein S15a, putative |
| Tb927.3.4370 | Tb927_03_v4: 1,224,042 - 1,225,736 | hypothetical protein, conserved |
| Tb927.3.4380 | Tb927_03_v4: 1,226,005 - 1,227,444 | hypothetical protein, conserved |
| Tb927.3.4390 | Tb927_03_v4: 1,227,814 - 1,229,331 | dihydrolipoamide dehydrogenase, putative |
| Tb927.3.4393 | Tb927_03_v4: 1,233,524 - 1,233,604 | tRNA Serine |
| Tb927.3.4396 | Tb927_03_v4: 1,233,810 - 1,233,891 | tRNA Leucine |
| Tb927.3.4399 | Tb927_03_v4: 1,233,951 - 1,234,031 | tRNA Serine |
| Tb927.3.4400 | Tb927_03_v4: 1,239,476 - 1,240,639 | hypothetical protein, conserved |
| Tb927.3.4410 | Tb927_03_v4: 1,240,825 - 1,241,487 | hypothetical protein, conserved |
| Tb927.3.4420 | Tb927_03_v4: 1,241,845 - 1,243,176 | hypothetical protein, conserved |
| Tb927.3.4430 | Tb927_03_v4: 1,243,455 - 1,245,281 | hypothetical protein, conserved |
| Tb927.3.4440 | Tb927_03_v4: 1,245,829 - 1,246,869 | hypothetical protein, conserved |
| Tb927.3.4450 | Tb927_03_v4: 1,247,753 - 1,248,361 | hypothetical protein, conserved |
| Tb927.3.4460 | Tb927_03_v4: 1,248,831 - 1,249,997 | hypothetical protein, conserved |
| Tb927.3.4470 | Tb927_03_v4: 1,251,153 - 1,252,631 | hypothetical protein, conserved |
| Tb927.3.4480 | Tb927_03_v4: 1,253,025 - 1,253,471 | hypothetical protein, conserved |
| Tb927.3.4490 | Tb927_03_v4: 1,254,394 - 1,256,232 | protein farnesyltransferase alpha subunit, putative |
| Tb927.3.4500 | Tb927_03_v4: 1,257,999 - 1,259,696 | fumarate hydratase, putative |
| Tb927.3.4510 | Tb927_03_v4: 1,260,457 - 1,264,452 | hypothetical protein, conserved |
| Tb927.3.4520 | Tb927_03_v4: 1,265,164 - 1,266,018 | hypothetical protein, conserved |
| Tb927.3.4530 | Tb927_03_v4: 1,266,277 - 1,267,044 | hypothetical protein, conserved |
| Tb927.3.4540 | Tb927_03_v4: 1,267,258 - 1,268,748 | hypothetical protein, conserved |
| Tb927.3.4550 | Tb927_03_v4: 1,269,073 - 1,271,100 | hypothetical protein, conserved |
| Tb927.3.4560 | Tb927_03_v4: 1,273,860 - 1,275,755 | serine/threonine protein kinase, putative |
| Tb927.3.4570 | Tb927_03_v4: 1,276,960 - 1,279,122 | N-acetylglucosaminyltransferase, putative |
| Tb927.3.4580 | Tb927_03_v4: 1,280,123 - 1,282,708 | hypothetical protein, conserved |
| Tb927.3.4590 | Tb927_03_v4: 1,287,351 - 1,288,268 | hypothetical protein, conserved |
| Tb927.3.4600 | Tb927_03_v4: 1,289,776 - 1,293,015 | hypothetical protein, conserved |
| Tb927.3.4610 | Tb927_03_v4: 1,293,531 - 1,302,836 | hypothetical protein, conserved |
| Tb927.3.4620 | Tb927_03_v4: 1,303,395 - 1,307,639 | hypothetical protein, conserved |
| Tb927.3.4630 | Tb927_03_v4: 1,308,545 - 1,313,572 | UDP-glucose:glycoprotein glucosyltransferase, putative |
| Tb927.3.4640 | Tb927_03_v4: 1,315,276 - 1,316,112 | hypothetical protein, conserved |
| Tb927.3.4650 | Tb927_03_v4: 1,317,239 - 1,317,904 | C-8 sterol isomerase, putative |
| Tb927.3.4660 | Tb927_03_v4: 1,318,525 - 1,320,315 | hypothetical protein, conserved |
| Tb927.3.4670 | Tb927_03_v4: 1,321,695 - 1,322,981 | cdc2-related kinase, putative |
| Tb927.3.4680 | Tb927_03_v4: 1,323,976 - 1,325,313 | RAB GDP dissociation inhibitor alpha, putative |
| Tb927.3.4690 | Tb927_03_v4: 1,325,946 - 1,327,439 | hypothetical protein, conserved |
| Tb927.3.4700 | Tb927_03_v4: 1,327,586 - 1,328,773 | hypothetical protein, conserved |
| Tb927.3.4710 | Tb927_03_v4: 1,330,991 - 1,332,625 | hypothetical protein, conserved |
| Tb927.3.4720 | Tb927_03_v4: 1,334,486 - 1,336,468 | dynamin, putative |
| Tb927.3.4730 | Tb927_03_v4: 1,336,737 - 1,337,888 | hypothetical protein, conserved; leucine-rich repeat protein (LRRP), putative |
| Tb927.3.4740 | Tb927_03_v4: 1,338,096 - 1,339,370 | hypothetical protein, conserved |
| Tb927.3.4750 | Tb927_03_v4: 1,339,741 - 1,342,356 | aminopeptidase, putative |
| Tb927.3.4760 | Tb927_03_v4: 1,344,510 - 1,346,492 | dynamin, putative |
| Tb927.3.4770 | Tb927_03_v4: 1,346,761 - 1,347,912 | hypothetical protein, conserved; leucine-rich repeat protein (LRRP), putative |
| Tb927.3.4780 | Tb927_03_v4: 1,348,120 - 1,349,394 | hypothetical protein, conserved |
| Tb927.3.4790 | Tb927_03_v4: 1,349,765 - 1,352,380 | aminopeptidase, putative |
| Tb927.3.4800 | Tb927_03_v4: 1,352,639 - 1,354,498 | hypothetical protein, conserved |
| Tb927.3.4810 | Tb927_03_v4: 1,354,950 - 1,355,801 | hypothetical protein, conserved |
| Tb927.3.4820 | Tb927_03_v4: 1,357,383 - 1,358,300 | acyltransferase, putative |
| Tb927.3.4830 | Tb927_03_v4: 1,360,573 - 1,361,247 | hypothetical protein |
| Tb927.3.4840 | Tb927_03_v4: 1,362,003 - 1,364,198 | ubiquitin hydrolase, putative |
| Tb927.3.4850 | Tb927_03_v4: 1,365,572 - 1,366,375 | enoyl-CoA hydratase, mitochondrial precursor, putative |
| Tb927.3.4860 | Tb927_03_v4: 1,368,289 - 1,369,407 | protein kinase, putative |
| Tb927.3.4870 | Tb927_03_v4: 1,369,724 - 1,370,764 | hypothetical protein, conserved |
| Tb927.3.4880 | Tb927_03_v4: 1,372,191 - 1,372,739 | hypothetical protein, conserved |
| Tb927.3.4890 | Tb927_03_v4: 1,372,947 - 1,373,561 | ubiquinone biosynthesis protein COQ7 homolog, putative |
| Tb927.3.4900 | Tb927_03_v4: 1,376,060 - 1,377,436 | tRNA isopentenyltransferase, putative |
| Tb927.3.4910 | Tb927_03_v4: 1,377,662 - 1,378,720 | signal peptide peptidase, putative |
| Tb927.3.4920 | Tb927_03_v4: 1,379,439 - 1,380,575 | hypothetical protein, conserved |
| Tb927.3.4930 | Tb927_03_v4: 1,382,459 - 1,383,085 | hypothetical protein, conserved |
| Tb927.3.4940 | Tb927_03_v4: 1,383,312 - 1,385,561 | hypothetical protein, conserved |
| Tb927.3.4950 | Tb927_03_v4: 1,387,078 - 1,388,502 | hypothetical protein, conserved |
| Tb927.3.4960 | Tb927_03_v4: 1,389,072 - 1,393,856 | kinesin, putative |
| Tb927.3.4970 | Tb927_03_v4: 1,395,382 - 1,398,573 | hypothetical protein, conserved |
| Tb927.3.4980 | Tb927_03_v4: 1,399,310 - 1,400,962 | hypothetical protein, conserved (pseudogene); hypothetical protein, conserved, frameshift |
| Tb927.3.4990 | Tb927_03_v4: 1,402,256 - 1,403,275 | oxidoreductase, putative |
| Tb927.3.5000 | Tb927_03_v4: 1,404,022 - 1,405,722 | hypothetical protein, conserved |
| Tb927.3.5010 | Tb927_03_v4: 1,406,292 - 1,406,873 | hypothetical protein, conserved |
| Tb927.3.5020 | Tb927_03_v4: 1,408,124 - 1,412,365 | hypothetical protein, conserved |
| Tb927.3.5030 | Tb927_03_v4: 1,412,986 - 1,415,199 | KU70 protein |
| Tb927.3.5040 | Tb927_03_v4: 1,415,779 - 1,417,473 | hypothetical protein, conserved |
| Tb927.3.5050 | Tb927_03_v4: 1,417,717 - 1,418,841 | 60S ribosomal protein L4 |
| Tb927.3.5060 | Tb927_03_v4: 1,419,145 - 1,420,188 | hypothetical protein, conserved |
| Tb927.3.5070 | Tb927_03_v4: 1,421,350 - 1,423,176 | hypothetical protein, conserved |
| Tb927.3.5080 | Tb927_03_v4: 1,424,370 - 1,425,965 | hypothetical protein, conserved |
| Tb927.3.5090 | Tb927_03_v4: 1,429,044 - 1,429,640 | tryparedoxin, putative |
| Tb927.3.5100 | Tb927_03_v4: 1,430,609 - 1,433,425 | DNA repair helicase and transcription factor protein, putative |
| Tb927.3.5110 | Tb927_03_v4: 1,433,835 - 1,435,292 | hypothetical protein, conserved |
| Tb927.3.5120 | Tb927_03_v4: 1,435,684 - 1,437,804 | hypothetical protein, conserved |
| Tb927.3.5130 | Tb927_03_v4: 1,438,142 - 1,439,500 | hypothetical protein, conserved |
| Tb927.3.5140 | Tb927_03_v4: 1,441,259 - 1,443,547 | hypothetical protein, conserved |
| Tb927.3.5150 | Tb927_03_v4: 1,446,003 - 1,446,878 | exonuclease, putative |
| Tb927.3.5160 | Tb927_03_v4: 1,447,852 - 1,451,550 | hypothetical protein, conserved |
| Tb927.3.5170 | Tb927_03_v4: 1,452,106 - 1,453,389 | hypothetical protein, conserved |
| Tb927.3.5180 | Tb927_03_v4: 1,453,906 - 1,454,316 | cofilin/actin depolymerizing factor, putative |
| Tb927.3.5190 | Tb927_03_v4: 1,456,977 - 1,458,053 | hypothetical protein, conserved |
| Tb927.3.5200 | Tb927_03_v4: 1,459,489 - 1,460,199 | hypothetical protein, conserved |
| Tb927.3.1780 | Tb927_03_v4: 462,143 - 462,532 | U6 snRNA-associated Sm-like protein LSm8p |
| Tb927.3.1790 | Tb927_03_v4: 464,118 - 465,164 | pyruvate dehydrogenase E1 beta subunit, putative |
| Tb927.3.1800 | Tb927_03_v4: 465,708 - 470,093 | hypothetical protein, conserved |
| Tb927.3.1810 | Tb927_03_v4: 470,816 - 471,796 | hypothetical protein, conserved |
| Tb927.3.1820 | Tb927_03_v4: 472,807 - 473,544 | hypothetical protein, conserved |
| Tb927.3.1830 | Tb927_03_v4: 474,527 - 475,954 | hypothetical protein, conserved |
| Tb927.3.1840 | Tb927_03_v4: 478,182 - 479,078 | 3-oxo-5-alpha-steroid 4-dehydrogenase, putative |
| Tb927.3.1850 | Tb927_03_v4: 480,261 - 482,048 | hypothetical protein, conserved |
| Tb927.3.1860 | Tb927_03_v4: 483,930 - 486,245 | hypothetical protein, conserved |
| Tb927.3.1870 | Tb927_03_v4: 487,184 - 488,053 | hypothetical protein, conserved |
| Tb927.3.1880 | Tb927_03_v4: 488,404 - 490,431 | hypothetical protein, conserved |
| Tb927.3.1890 | Tb927_03_v4: 490,953 - 491,807 | hypothetical protein, conserved |
| Tb927.3.1900 | Tb927_03_v4: 493,734 - 496,337 | hypothetical protein, conserved |
| Tb927.3.1891 | Tb927_03_v4: 494,224 - 494,301 | C/D snoRNA, TB3C2C1 |
| Tb927.3.1892 | Tb927_03_v4: 494,356 - 494,433 | C/D snoRNA, TB3C2C1 |
| Tb927.3.1893 | Tb927_03_v4: 494,488 - 494,539 | C/D snoRNA, TB3C2C1 |
| Tb927.3.1894 | Tb927_03_v4: 494,596 - 494,673 | C/D snoRNA, TB3C2C1 |
| Tb927.3.1895 | Tb927_03_v4: 494,728 - 494,805 | C/D snoRNA, TB3C2C1 |
| Tb927.3.1896 | Tb927_03_v4: 494,860 - 494,965 | C/D snoRNA, TB3C2C1 |
| Tb927.3.1905 | Tb927_03_v4: 495,034 - 495,139 | C/D snoRNA, TB3C2C1 |
| Tb927.3.1910 | Tb927_03_v4: 500,960 - 502,735 | hypothetical protein, conserved |
| Tb927.3.1920 | Tb927_03_v4: 505,714 - 507,528 | hypothetical protein, conserved |
| Tb927.3.1930 | Tb927_03_v4: 508,342 - 509,781 | hypothetical protein, conserved |
| Tb927.3.1940 | Tb927_03_v4: 510,641 - 511,957 | hypothetical protein, conserved |
| Tb927.3.1960 | Tb927_03_v4: 515,152 - 515,589 | hypothetical protein, conserved |
| Tb927.3.1970 | Tb927_03_v4: 516,166 - 517,134 | hypothetical protein, conserved |
| Tb927.3.1980 | Tb927_03_v4: 517,473 - 518,363 | hypothetical protein, conserved |
| Tb927.3.1990 | Tb927_03_v4: 519,226 - 520,671 | hypothetical protein, conserved |
| Tb927.3.2000 | Tb927_03_v4: 521,498 - 522,151 | hypothetical protein, conserved |
| Tb927.3.2010 | Tb927_03_v4: 522,585 - 524,333 | hypothetical protein, conserved |
| Tb927.3.2020 | Tb927_03_v4: 525,009 - 527,249 | kinesin, putative |
| Tb927.3.2030 | Tb927_03_v4: 527,847 - 528,179 | acylphosphatase, putative |
| Tb927.3.2040 | Tb927_03_v4: 529,310 - 532,528 | kinesin, putative |
| Tb927.3.2050 | Tb927_03_v4: 534,327 - 539,045 | hypothetical protein, conserved |
| Tb927.3.2060 | Tb927_03_v4: 541,020 - 542,327 | protein kinase, putative |
| Tb927.3.2070 | Tb927_03_v4: 543,223 - 547,281 | hypothetical protein, conserved |
| Tb927.3.2080 | Tb927_03_v4: 548,257 - 549,039 | hypothetical protein, conserved |
| Tb927.3.2090 | Tb927_03_v4: 550,052 - 551,899 | aminopeptidase P1, putative |
| Tb927.3.2100 | Tb927_03_v4: 553,025 - 553,951 | hypothetical protein, conserved |
| Tb927.3.2110 | Tb927_03_v4: 556,358 - 557,629 | TFIIF-stimulated CTD phosphatase, putative |
| Tb927.3.2120 | Tb927_03_v4: 558,218 - 559,216 | hypothetical protein, conserved |
| Tb927.3.2130 | Tb927_03_v4: 560,389 - 562,875 | hypothetical protein, conserved |
| Tb927.3.2140 | Tb927_03_v4: 563,494 - 566,340 | transcription activator, putative |
| Tb927.3.2150 | Tb927_03_v4: 566,856 - 567,944 | protein phosphatase 2C, putative |
| Tb927.3.2160 | Tb927_03_v4: 568,373 - 569,776 | hypothetical protein, conserved |
| Tb927.3.2170 | Tb927_03_v4: 570,057 - 572,729 | translation elongation factor EF-2, putative |
| Tb927.3.2180 | Tb927_03_v4: 573,648 - 574,118 | hypothetical protein, conserved |
| Tb927.3.2190 | Tb927_03_v4: 574,447 - 575,208 | RNA triphosphatase |
| Tb927.3.2200 | Tb927_03_v4: 575,548 - 576,594 | hypothetical protein, conserved |
| Tb927.3.2210 | Tb927_03_v4: 577,319 - 579,103 | hypothetical protein, conserved |
| Tb927.3.2220 | Tb927_03_v4: 579,399 - 580,061 | hypothetical protein, conserved |
| Tb927.3.2230 | Tb927_03_v4: 580,931 - 581,836 | succinyl-CoA synthetase alpha subunit, putative |
| Tb927.3.2240 | Tb927_03_v4: 582,246 - 583,445 | hypothetical protein, conserved |
| Tb927.3.2250 | Tb927_03_v4: 583,617 - 584,513 | hypothetical protein, conserved |
| Tb927.3.2260 | Tb927_03_v4: 584,707 - 585,393 | hypothetical protein, conserved |
| Tb927.3.2270 | Tb927_03_v4: 587,858 - 589,183 | hypothetical protein, conserved |
| Tb927.3.2280 | Tb927_03_v4: 589,443 - 591,236 | vacuolar sorting protein 33 , putative |
| Tb927.3.2290 | Tb927_03_v4: 591,399 - 592,520 | chaperone protein DNAJ, putative |
| Tb927.3.2300 | Tb927_03_v4: 592,647 - 593,252 | hypothetical protein, conserved |
| Tb927.3.2310 | Tb927_03_v4: 593,475 - 594,377 | flagellar component |
| Tb927.3.2320 | Tb927_03_v4: 594,909 - 596,231 | COP9 signalosome complex subunit 2, putative |
| Tb927.3.2330 | Tb927_03_v4: 597,683 - 599,539 | hypothetical protein, conserved |
| Tb927.3.2340 | Tb927_03_v4: 599,846 - 600,844 | peroxin-2 |
| Tb927.3.2350 | Tb927_03_v4: 601,321 - 601,842 | hypothetical protein, conserved |
| Tb927.3.2360 | Tb927_03_v4: 602,105 - 603,532 | hypothetical protein, conserved |
| Tb927.3.2370 | Tb927_03_v4: 603,856 - 604,260 | hypothetical protein, conserved |
| Tb927.3.2380 | Tb927_03_v4: 604,765 - 605,085 | hypothetical protein, conserved |
| Tb927.3.2390 | Tb927_03_v4: 605,628 - 606,956 | hypothetical protein, conserved |
| Tb927.3.2400 | Tb927_03_v4: 607,321 - 608,244 | hypothetical protein, conserved |
| Tb927.3.2410 | Tb927_03_v4: 608,797 - 609,693 | peroxisome assembly protein, putative |
| Tb927.3.2420 | Tb927_03_v4: 610,112 - 610,492 | hypothetical protein, conserved |
| Tb927.3.2430 | Tb927_03_v4: 610,862 - 613,624 | hypothetical protein, conserved |
| Tb927.3.2440 | Tb927_03_v4: 614,482 - 615,702 | serine/threonine-protein kinase, putative |
| Tb927.3.2450 | Tb927_03_v4: 616,993 - 617,490 | hypothetical protein, conserved |
| Tb927.3.2460 | Tb927_03_v4: 617,808 - 618,815 | hypothetical protein, conserved |
| Tb927.3.2470 | Tb927_03_v4: 619,537 - 621,351 | RNA-binding protein, putative; pumillio RNA binding protein 8, putative |
| Tb927.3.2480 | Tb927_03_v4: 625,140 - 625,898 | hypothetical protein, conserved |
| Tb927.3.2490 | Tb927_03_v4: 626,648 - 629,539 | hypothetical protein, conserved |
| Tb927.3.2500 | Tb927_03_v4: 631,198 - 632,385 | hypothetical protein |
| Tb927.3.2510 | Tb927_03_v4: 632,832 - 634,202 | expression site-associated gene (ESAG) protein, putative |
| Tb927.3.2520 | Tb927_03_v4: 635,419 - 636,393 | expression site-associated gene (ESAG) protein, putative |
| Tb927.3.2530 | Tb927_03_v4: 636,520 - 637,371 | expression site-associated gene (ESAG, pseudogene), putative |
| Tb927.3.2540 | Tb927_03_v4: 637,733 - 638,956 | variant surface glycoprotein (VSG)-related, putative |
| Tb927.3.2550 | Tb927_03_v4: 640,907 - 641,563 | hypothetical protein, conserved |
| Tb927.3.2560 | Tb927_03_v4: 642,421 - 643,077 | hypothetical protein, conserved |
| Tb927.3.2570 | Tb927_03_v4: 643,935 - 644,591 | hypothetical protein, conserved |
| Tb927.3.2580 | Tb927_03_v4: 645,449 - 646,204 | hypothetical protein, conserved |
| Tb927.3.2590 | Tb927_03_v4: 647,032 - 647,949 | hypothetical protein |
| Tb927.3.2600 | Tb927_03_v4: 652,522 - 659,034 | ATP-dependent DEAD/H RNA helicase, putative |
| Tb927.3.2610 | Tb927_03_v4: 659,787 - 662,132 | hypothetical protein, conserved |
| Tb927.3.2620 | Tb927_03_v4: 662,834 - 668,887 | hypothetical protein, conserved |
| Tb927.3.2630 | Tb927_03_v4: 670,204 - 672,552 | hypothetical protein, conserved |
| Tb927.3.2640 | Tb927_03_v4: 673,016 - 674,149 | hypothetical protein, conserved |
| Tb927.3.2650 | Tb927_03_v4: 674,715 - 674,936 | cytochrome c oxidase copper chaperone, putative |
| Tb927.3.2660 | Tb927_03_v4: 675,209 - 677,398 | hypothetical protein, conserved |
| Tb927.3.2670 | Tb927_03_v4: 677,840 - 678,649 | hypothetical protein, conserved |
| Tb927.3.2680 | Tb927_03_v4: 679,159 - 680,754 | hypothetical protein, conserved |
| Tb927.3.2690 | Tb927_03_v4: 682,253 - 686,038 | serine/threonine-protein kinase, putative |
| Tb927.3.2700 | Tb927_03_v4: 687,015 - 688,181 | hypothetical protein, conserved |
| Tb927.3.2710 | Tb927_03_v4: 689,701 - 692,772 | 6-phosphofructo-2-kinase/fructose-2,6-biphospha ta se,putative |
| Tb927.3.2720 | Tb927_03_v4: 693,756 - 694,697 | hypothetical protein |
| Tb927.3.2730 | Tb927_03_v4: 695,723 - 700,807 | hypothetical protein, conserved |
| Tb927.3.2740 | Tb927_03_v4: 701,653 - 702,573 | hypothetical protein, conserved |
| Tb927.3.2750 | Tb927_03_v4: 703,304 - 705,919 | hypothetical protein, conserved |
| Tb927.3.2760 | Tb927_03_v4: 706,555 - 711,231 | hypothetical protein, conserved |
| Tb927.3.2770 | Tb927_03_v4: 711,932 - 715,342 | hypothetical protein, conserved |
| Tb927.3.2780 | Tb927_03_v4: 716,118 - 719,426 | hypothetical protein, conserved |
| Tb927.3.2790 | Tb927_03_v4: 720,110 - 723,544 | hypothetical protein, conserved; leucine-rich repeat protein (LRRP), putative |
| Tb927.3.2800 | Tb927_03_v4: 724,157 - 726,163 | hypothetical protein |
| Tb927.3.2810 | Tb927_03_v4: 726,539 - 731,566 | hypothetical protein, conserved |
| Tb927.3.2820 | Tb927_03_v4: 732,032 - 733,735 | TFIIF-stimulated CTD phosphatase, putative |
| Tb927.3.2830 | Tb927_03_v4: 734,433 - 735,527 | hypothetical protein, conserved |
| Tb927.3.2840 | Tb927_03_v4: 735,988 - 736,773 | inorganic pyrophosphatase, putative |
| Tb927.3.2850 | Tb927_03_v4: 737,199 - 738,035 | hypothetical protein, conserved |
| Tb927.3.2860 | Tb927_03_v4: 738,680 - 739,582 | hypothetical protein, conserved |
| Tb927.3.2870 | Tb927_03_v4: 740,401 - 742,365 | hypothetical protein, conserved |
| Tb927.3.2880 | Tb927_03_v4: 743,318 - 743,632 | hypothetical protein, conserved |
| Tb927.3.2890 | Tb927_03_v4: 744,287 - 745,255 | radial spoke protein RSP10, putative |
| Tb927.3.2900 | Tb927_03_v4: 746,906 - 748,162 | elongation initiation factor 2 alpha subunit, putative |
| Tb927.3.2910 | Tb927_03_v4: 749,536 - 749,946 | hypothetical protein, conserved |
| Tb927.3.2920 | Tb927_03_v4: 750,804 - 751,811 | hypothetical protein, conserved |
| Tb927.3.2930 | Tb927_03_v4: 756,578 - 757,297 | RNA-binding protein RBP6, putative |
| Tb927.3.2940 | Tb927_03_v4: 758,484 - 759,392 | hypothetical protein, conserved |
| Tb927.3.2950 | Tb927_03_v4: 760,473 - 762,752 | hypothetical protein, conserved; leucine-rich repeat protein (LRRP), putative; ribonuclease inhibitor- like protein |
| Tb927.3.2960 | Tb927_03_v4: 763,597 - 764,580 | inosine-adenosine-guanosine-nucleosidehydrolase |
| Tb927.3.2970 | Tb927_03_v4: 764,994 - 765,584 | hypothetical protein, conserved |
| Tb927.3.2980 | Tb927_03_v4: 766,457 - 767,326 | mitochondrial carrier protein, putative |
| Tb927.3.2990 | Tb927_03_v4: 767,838 - 768,446 | hypothetical protein, conserved |
| Tb927.3.3000 | Tb927_03_v4: 769,604 - 771,304 | flagellar transport protein, putative |
| Tb927.3.3010 | Tb927_03_v4: 771,764 - 771,988 | hypothetical protein, conserved |
| Tb927.3.3020 | Tb927_03_v4: 772,495 - 773,475 | actin-like protein, putative |
| Tb927.3.3030 | Tb927_03_v4: 774,600 - 775,709 | hypothetical protein, conserved |
| Tb927.3.3040 | Tb927_03_v4: 777,462 - 778,265 | hypothetical protein, conserved |
| Tb927.3.3050 | Tb927_03_v4: 778,755 - 781,850 | hypothetical protein, conserved |
| Tb927.3.3060 | Tb927_03_v4: 784,857 - 785,771 | hypothetical protein, conserved |
| Tb927.3.3070 | Tb927_03_v4: 787,225 - 789,954 | 3', 5'-cyclic nucleotide phosphodiesterase, putative |
| Tb927.3.3080 | Tb927_03_v4: 790,605 - 792,107 | serine/threonine-protein kinase NEK1, putative |
| Tb927.3.3090 | Tb927_03_v4: 792,432 - 795,323 | helicase, putative |
| Tb927.3.3100 | Tb927_03_v4: 795,971 - 796,339 | peptidyl-prolyl cis-trans isomerase NIMA-interacting 4, putative |
| Tb927.3.3110 | Tb927_03_v4: 796,976 - 800,161 | hypothetical protein, conserved |
| Tb927.3.3120 | Tb927_03_v4: 800,708 - 802,009 | hypothetical protein, conserved |
| Tb927.3.3130 | Tb927_03_v4: 803,274 - 808,202 | hypothetical protein, conserved |
| Tb927.3.3140 | Tb927_03_v4: 809,070 - 809,465 | hypothetical protein, conserved |
| Tb927.3.3150 | Tb927_03_v4: 809,741 - 812,098 | hypothetical protein, conserved |
| Tb927.3.3160 | Tb927_03_v4: 813,206 - 815,319 | poly(A) polymerase |
| Tb927.3.3170 | Tb927_03_v4: 815,897 - 817,861 | hypothetical protein, conserved |
| Tb927.3.3180 | Tb927_03_v4: 818,610 - 821,588 | hypothetical protein, conserved |
| Tb927.3.3190 | Tb927_03_v4: 823,161 - 824,672 | serine/threonine-protein kinase, putative |
| Tb927.3.3200 | Tb927_03_v4: 827,057 - 828,253 | hypothetical protein, conserved |
| Tb927.3.3210 | Tb927_03_v4: 828,696 - 829,235 | hypothetical protein |
| Tb927.3.3220 | Tb927_03_v4: 830,050 - 832,755 | hypothetical protein, conserved |
| Tb927.3.3230 | Tb927_03_v4: 833,221 - 835,050 | hypothetical protein, conserved |
| Tb927.3.3240 | Tb927_03_v4: 835,695 - 835,919 | hypothetical protein, conserved |
| Tb927.3.3250 | Tb927_03_v4: 836,366 - 837,409 | hypothetical protein, conserved |
| Tb927.3.3260 | Tb927_03_v4: 837,686 - 838,654 | hypothetical protein, conserved |
| Tb927.3.3270 | Tb927_03_v4: 840,818 - 842,281 | ATP-dependent phosphofructokinase |
| Tb927.3.3280 | Tb927_03_v4: 843,271 - 844,605 | katanin, putative; serine peptidase, Clan SJ, family S16, putative |
| Tb927.3.3290 | Tb927_03_v4: 845,530 - 846,948 | protein kinase, putative |
| Tb927.3.3300 | Tb927_03_v4: 848,039 - 850,483 | hypothetical protein, conserved |
| Tb927.3.3310 | Tb927_03_v4: 850,898 - 851,587 | 60S ribosomal protein L13, putative |
| Tb927.3.3320 | Tb927_03_v4: 851,801 - 852,457 | 60S ribosomal protein L13, putative |
| Tb927.3.3330 | Tb927_03_v4: 853,025 - 853,450 | heat shock protein 20, putative |
| Tb927.3.3340 | Tb927_03_v4: 855,193 - 857,223 | 3', 5'-cyclic nucleotide phosphodiesterase, putative |
| Tb927.3.3350 | Tb927_03_v4: 858,697 - 860,448 | hypothetical protein, conserved |
| Tb927.3.3360 | Tb927_03_v4: 860,667 - 862,067 | acyltransferase, putative |
| Tb927.3.3370 | Tb927_03_v4: 862,437 - 863,786 | hypothetical protein, conserved |
| Tb927.3.3380 | Tb927_03_v4: 865,111 - 866,205 | TFIIF-stimulated CTD phosphatase, putative |
| Tb927.3.3390 | Tb927_03_v4: 866,616 - 868,391 | kinesin, putative |
| Tb927.3.3400 | Tb927_03_v4: 868,667 - 870,403 | kinesin, putative |
| Tb927.3.3410 | Tb927_03_v4: 871,164 - 872,519 | aspartyl aminopeptidase, putative |
| Tb927.3.3420 | Tb927_03_v4: 895,831 - 896,616 | hypothetical protein (pseudogene); hypothetical protein, point mutation |
| Tb927.3.3421 | Tb927_03_v4: 902,861 - 905,111 | rRNA small subunit |
| Tb927.3.3422 | Tb927_03_v4: 905,452 - 905,623 | rRNA 5.8S (M3) |
| Tb927.3.3423 | Tb927_03_v4: 906,206 - 908,032 | rRNA large subunit alpha |
| Tb927.3.3424 | Tb927_03_v4: 908,228 - 908,442 | rRNA large subunit gamma (M1) |
| Tb927.3.3425 | Tb927_03_v4: 908,519 - 910,003 | rRNA large subunit beta |
| Tb927.3.3426 | Tb927_03_v4: 910,141 - 910,323 | rRNA large subunit delta (M2) |
| Tb927.3.3427 | Tb927_03_v4: 910,655 - 910,732 | rRNA large subunit zeta (M6) |
| Tb927.3.3428 | Tb927_03_v4: 911,053 - 911,188 | rRNA large subunit epsilon (M4) |
| Tb927.3.3429 | Tb927_03_v4: 921,223 - 923,473 | rRNA small subunit |
| Tb927.3.3431 | Tb927_03_v4: 923,814 - 923,985 | rRNA 5.8S (M3) |
| Tb927.3.3432 | Tb927_03_v4: 924,568 - 926,394 | rRNA large subunit alpha |
| Tb927.3.3433 | Tb927_03_v4: 926,590 - 926,804 | rRNA large subunit gamma (M1) |
| Tb927.3.3434 | Tb927_03_v4: 926,881 - 928,365 | rRNA large subunit beta |
| Tb927.3.3435 | Tb927_03_v4: 928,503 - 928,685 | rRNA large subunit delta (M2) |
| Tb927.3.3436 | Tb927_03_v4: 929,008 - 929,085 | rRNA large subunit zeta (M6) |
| Tb927.3.3437 | Tb927_03_v4: 929,408 - 929,543 | rRNA large subunit epsilon (M4) |
| Tb927.3.3438 | Tb927_03_v4: 939,578 - 941,828 | rRNA small subunit |
| Tb927.3.3439 | Tb927_03_v4: 942,169 - 942,340 | rRNA 5.8S (M3) |
| Tb927.3.3441 | Tb927_03_v4: 942,923 - 944,749 | rRNA large subunit alpha |
| Tb927.3.3442 | Tb927_03_v4: 944,945 - 945,159 | rRNA large subunit gamma (M1) |
| Tb927.3.3443 | Tb927_03_v4: 945,236 - 946,720 | rRNA large subunit beta |
| Tb927.3.3444 | Tb927_03_v4: 946,858 - 947,040 | rRNA large subunit delta (M2) |
| Tb927.3.3445 | Tb927_03_v4: 947,368 - 947,445 | rRNA large subunit zeta (M6) |
| Tb927.3.3446 | Tb927_03_v4: 947,768 - 947,903 | rRNA large subunit epsilon (M4) |
| Tb927.3.3447 | Tb927_03_v4: 957,940 - 960,190 | rRNA small subunit |
| Tb927.3.3448 | Tb927_03_v4: 960,539 - 960,710 | rRNA 5.8S (M3) |
| Tb927.3.3449 | Tb927_03_v4: 961,295 - 963,123 | rRNA large subunit alpha |
| Tb927.3.3451 | Tb927_03_v4: 963,319 - 963,533 | rRNA large subunit gamma (M1) |
| Tb927.3.3452 | Tb927_03_v4: 963,610 - 965,094 | rRNA large subunit beta |
| Tb927.3.3453 | Tb927_03_v4: 965,232 - 965,414 | rRNA large subunit delta (M2) |
| Tb927.3.3454 | Tb927_03_v4: 965,735 - 965,812 | rRNA large subunit zeta (M6) |
| Tb927.3.3455 | Tb927_03_v4: 966,132 - 966,267 | rRNA large subunit epsilon (M4) |
| Tb927.3.3430 | Tb927_03_v4: 967,381 - 967,896 | hypothetical protein, conserved |
| Tb927.3.3440 | Tb927_03_v4: 977,888 - 978,925 | hypothetical protein, conserved |
| Tb927.3.3450 | Tb927_03_v4: 979,573 - 980,109 | ADP-ribosylation factor-like protein 3A, putative |
| Tb927.3.3470 | Tb927_03_v4: 982,589 - 983,131 | cytochrome b5, putative |
| Tb927.3.3480 | Tb927_03_v4: 983,655 - 984,008 | U2 small nuclear ribonucleoprotein B, putative |
| Tb927.3.3490 | Tb927_03_v4: 985,621 - 986,436 | high mobility group protein, putative |
| Tb927.3.3500 | Tb927_03_v4: 987,719 - 988,804 | hypothetical protein, conserved |
| Tb927.3.3510 | Tb927_03_v4: 989,200 - 990,675 | hypothetical protein, conserved |
| Tb927.3.3520 | Tb927_03_v4: 991,358 - 992,641 | hypothetical protein, conserved |
| Tb927.3.3530 | Tb927_03_v4: 993,259 - 994,611 | hypothetical protein, conserved |
| Tb927.3.3540 | Tb927_03_v4: 994,998 - 996,533 | hypothetical protein, conserved |
| Tb927.3.3550 | Tb927_03_v4: 996,854 - 1,000,345 | hypothetical protein, conserved |
